# Synthesis of Imidazo[2,1‑*a*]isoindolones via Rearrangement and Tandem Cyclization of Amino-Acid-Based *N*‑Phenacyl-2-cyano-4-nitrobenzensulfonamides

**DOI:** 10.1021/acs.joc.4c03113

**Published:** 2025-05-27

**Authors:** Barbora Lemrová, Kateřina Žáková, Miroslav Soural

**Affiliations:** Department of Organic Chemistry, Faculty of Science, 48207 Palacký University, Olomouc 771 46, Czech Republic

## Abstract

Esters of amino acids were reacted with 2-cyano-4-nitrobenzenesulfonyl chloride or 2-cyano-4-(trifluoromethyl)­benzenesulfonyl chloride and alkylated with various α-haloketones. The corresponding sulfonamides were heated in a solution of ammonium acetate, which yielded imidazo­[2,1-*a*]­isoindolones in one step. The key reaction was based on intramolecular C-arylation followed by spontaneous cycloaddition and cyclocondensation. Two approaches have been developed: (i) solid-phase synthesis starting from amino acids immobilized on Wang resin, which allows the rapid preparation of target compounds using the cyclative cleavage strategy, and (ii) traditional solution-phase synthesis using amino acid methyl esters as the starting materials. The advantages and drawbacks of both alternatives are compared.

## Graphical abstract

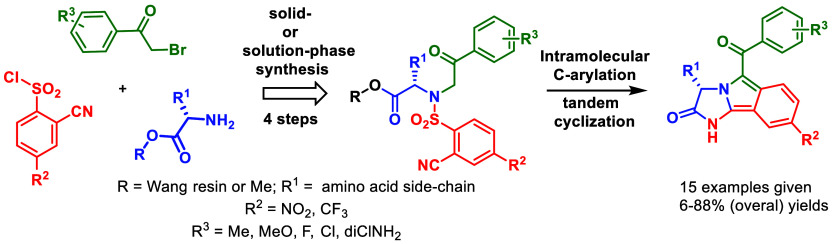

## Introduction

The Smiles-type rearrangement of substrates containing the combination of electron-poor aromatic rings with alkyl groups activated by adjacent electron-withdrawing functionalities is a simple strategy to create C­(sp^3^)–C­(sp^2^) bonds without the need for metal catalysis.^1,2^ Recently, this approach was applied for the synthesis of different heterocyclic scaffolds from various *N*-substituted nitrobenzenesulfonamides.^3^ The reaction proceeds *via* a spiro-Meisenheimer complex, which is spontaneously converted to the C-arylated product. After rearrangement, the nitro group at the *o*-position can serve as the electrophile for the spontaneous heterocyclization,^4,5^ whereas the use of *p*-nitro derivatives results in 4-nitrophenyl substitution of final heterocycles.^6–8^ Interestingly, only the nitro group was used as a suitable EWG in all reported heterocyclic applications. Inspired by the previously reported synthesis of indazol oxides,^9^ we attempted to replace *o*-nitro with nitrile to possibly prepare imidazo­[2,1-*a*]­isoindol-2­(3*H*)-ones (Figure 1).

**Figure 1:**
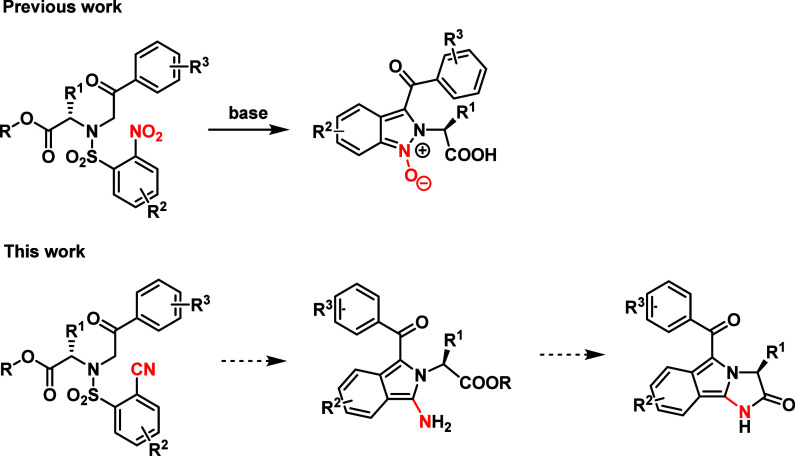
Design of the synthetic strategy eventually leading to imidazo­[2,1-*a*]­isoindol-2­(3*H*)-ones.

Isoindoles occur in alkaloids^10–13^ as well as synthetic products^14–18^ and have been reported as promising anticancer agents^19–24^ or compounds with antithrombotic,^11^ anti-TMV,^12^ anti-inflammatory,^25,26^ antiproliferative,^27–29^ and antibacterial^30,31^ activities. Furthermore, with respect to their beneficial spectral properties, isoindoles have been used as building blocks in the construction of BODIPY fluorophores^32,33^ or pigments^34–36^ (Figure 2). Consequently, different synthetic approaches for accessing fused isoindoles have been developed using cycloaddition,^14,37–40^ cyclocondensation,^11,22,29,41–44^ or metal-catalyzed couplings.^45,46^ In this article, we report an original approach leading to novel imidazo­[2,1-*a*]­isoindolones using either traditional solution-phase chemistry or solid-phase synthesis (SPS). Although originally developed for peptides, SPS has also been widely applied to the preparation of small molecules. The key advantage is the simple isolation of all reaction intermediates in the multistep sequence with a minimum hands-on time. For this reason, SPS has been utilized by the pharmaceutical industry to rapidly produce compounds via parallel synthesis.^47^ On the other hand, SPS is limited to rather lower quantities of compounds (excluding the production of peptides). For this reason, we also report protocols for traditional solution-phase alternatives.

**Figure 2:**
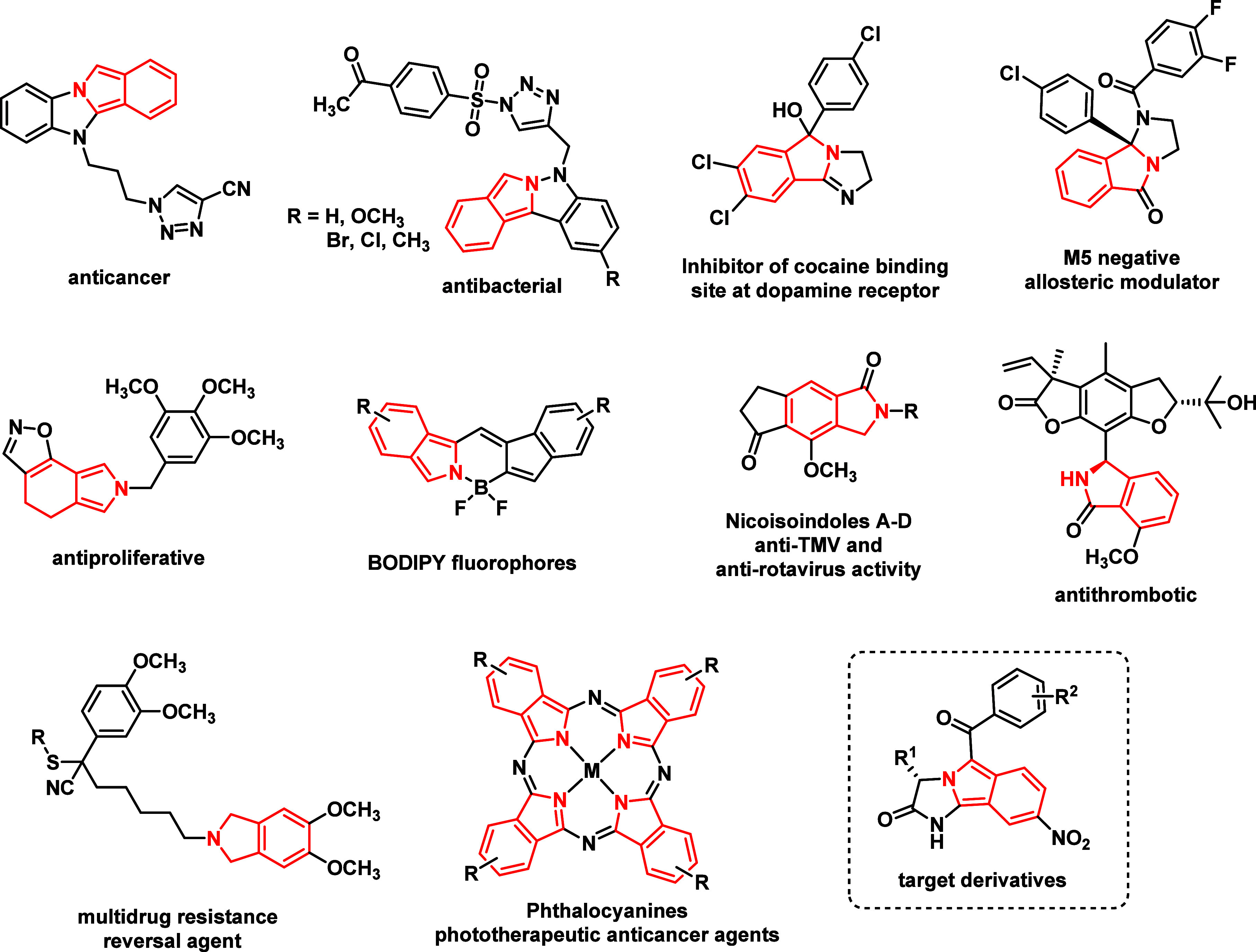
Isoindoles with interesting biological or physicochemical properties.

## Results and Discussion

To verify the feasibility of solid-supported alternatives, Wang resin **1** was acylated with Fmoc-Ala-OH followed by Fmoc cleavage, sulfonylation with 2-cyanobenzenesulfonyl chloride, and alkylation with 2-bromo-1-(*p*-tolyl)­ethan-1-one (Scheme 1). The intermediate **4a** (R^1^ = Me, R^2^ = H, R^3^ = Me) was smoothly obtained in an excellent crude purity of 91% (analyzed by UPLC after the acidic cleavage of the compound from the polymer support).

**Scheme 1:**
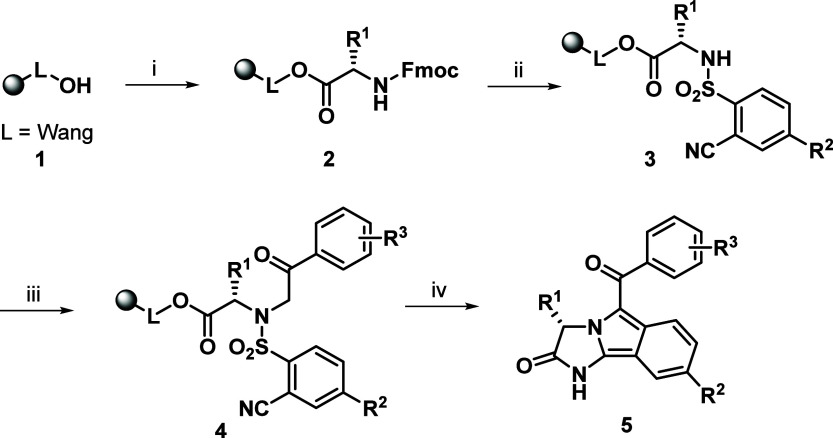
General Synthetic Approach to Imidazo­[2,1-*a*]­isoindol-2­(3*H*)-ones Using SPS^a^

a Reagents and conditions: (i) Fmoc-AA-OH, HOBt, DIC, DMAP, DMF/DMF (1/1), rt, 24 h; (ii) 20% piperidine in DMF, rt, 20 min then 2-cyanobenzenesulfonyl chloride or 2-cyano-4-nitrobenzenesulfonyl chloride, 2,6-lutidine, DCM, rt, on; (iii) α-bromoketone, DIPEA, DMF, rt, 4 h; and (iv) ammonium acetate, acetonitrile, water, 100 °C, 2 h.

The immobilized sulfonamide **4a** was reacted with different bases in various solvents under different conditions, including heating (data not shown); however, no conversion was observed, and only the starting material was obtained after cleavage from the solid support. This outcome showed that for the studied substrates, the cyano group itself is not a suitable functionality to provide the spiro-Meisenheimer complex. Consequently, 2-cyanobenzenesulfonyl chloride was replaced with 2-cyano-4-nitrobenzenesulfonyl chloride, which yielded immobilized sulfonamide **4b** (R^1^ = Me, R^2^ = NO_2_, and R^3^ = Me) with a crude purity of 94%. Its exposure to 0.2 M DBU/DMF, as in the case of previously reported indazol-oxides,^9^ led to the rapid disappearance of the starting material; however, only a mixture of unknown compounds was detected by UPLC-MS. For this reason, different conditions to control the reaction outcome were screened (see Table 1). Notably, the formation of the desired compound would lead to spontaneous release from the resin (see the mechanistic description later in the text); hence, it was necessary to analyze the resin-bound materials (upon acidic cleavage) as well as the reaction solution.

**Table 1:** Optimization of Reaction Conditions for the Conversion of Sulfonamide **4b** to **5b**

entry	reagent	conc. (M)	solvent	temp. (°C)	time	crude purity of **5b** (%)^a^
1	DBU	0.2	DMF	rt	1 h	0 (mixture of unkn. cmpds.)
2	DBU	0.06	DMF	rt	1 h	80 (low reproducibility)
3	DBU	0.06	ACN/H_2_O	rt	1 h	0 (mixture of unkn. cmpds.)
4	DBU	0.02	ACN/H_2_O	rt	1 h	0 (starting material)
5	TMSOK	0.08	DMF	rt	1 h	0 (mixture of unkn. cmpds.)
6	TMSOK	0.02	DMF	rt	1 h	0 (starting material)
7	TMSOK	0.2	ACN	rt	1 h	0 (starting material)
8	DIPEA	0.2	ACN	rt	1 h	0 (starting material)
9	DIPEA	0.5	DMF	rt	1 h	0 (starting material)
10	AmAc	0.2	ACN/H_2_O	rt	2 h	0 (starting material)
11	AmAc	0.2	ACN/H_2_O	100	2 h	85
12	AmAc	0.2	ACN	100	2 h	80
13	AcOH	0.2	ACN	100	2 h	30 (numerous byproducts)
14			ACN	100	2 h	6 (numerous byproducts)
15			ACN/H_2_O	100	2 h	35 (starting material)

a Crude purity calculated from UPLC-UV traces.

Further experiments revealed that the desired conversion can be accomplished with DBU/DMF, but it requires a lower concentration of the base. Whereas 0.2 M solution led to full decomposition, 0.06 M (i.e., only 1% DBU) yielded the final product in high crude purity. Nevertheless, this protocol suffers from low reproducibility and very problematic scale-up. For this reason, TMSOK in DMF was also tested as reported for similar substrates,^6^ but it did not work (see entries 5–7) as well as DIPEA.

Finally, a serendipitous discovery revealed that ammonium acetate can be used as a reagent, although it has not been previously reported for this type of conversion. We observed that the purification of intermediate **4b** via reverse-phase HPLC in ammonium acetate buffer led to slow conversion to **5b**. After careful optimization, heating in 0.2 M solution of ammonium acetate in acetonitrile/water was verified as the most robust and fully reproducible procedure.

The plausible reaction mechanism for the conversion of **4b** to **5b** is depicted in Scheme 2 and suggests that the cascade reactions involve several steps. First, ammonium acetate triggers the formation of spiro-Meisenheimer complex **A**,^48–50^ which rearranges to the C-arylated intermediate **B**. The spontaneous cycloaddition and aromatization to **D** are followed by the formation of **5b** *via* cyclocondensation. None of the suggested intermediates were detected under the developed conditions. Compound **5b** was obtained as a single product with a crude purity of 85% and was isolated in an overall yield of 60% (after three reaction steps, the yield was calculated from the loading of immobilized amino acid **2a**). The conversion of **D** to **5b** shows that the liberation of the final product from the polymer support takes place via the “cyclative cleavage”.^51,52^ This scenario positively influences the crude purity because immobilized byproducts of different structures remain attached to the resin and can be isolated by simple filtration.

**Scheme 2:**
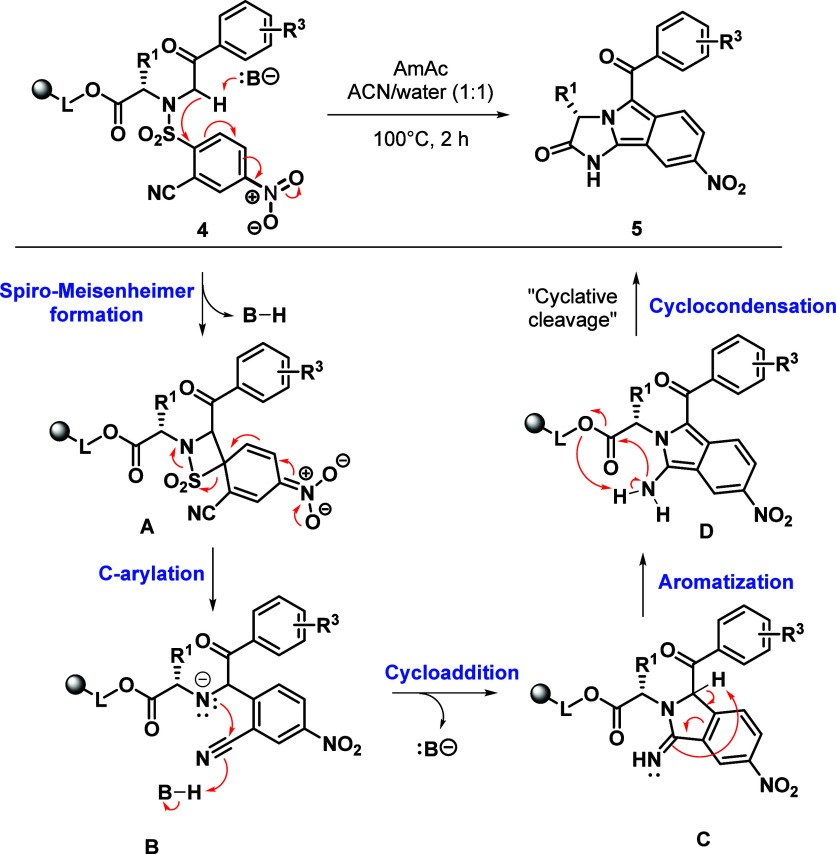
Proposed Reaction Mechanism

With optimized conditions in hand, we evaluated the scope and limitations of our method using different starting materials. Proteinogenic amino acids with variously substituted side chains, such as Fmoc-Ala-OH, Fmoc-Phe-OH, Fmoc-Lys­(Boc)-OH, Fmoc-Tyr­(*t*Bu)-OH, Fmoc-Glu­(O*t*Bu)-OH, Fmoc-Leu-OH, Fmoc-Trp­(Boc)-OH, and Fmoc-Arg­(Pbf)-OH, were used. Additionally, unnatural Fmoc-homoazidoalanin and Fmoc-β-Ala-OH were also used to synthesize homological dihydropyrimido­[2,1-*a*]­isoindol-2­(1*H*)-ones. In the case of bromoketones, derivatives with electron-withdrawing and -donating groups or their combination were selected. The list of building blocks is depicted in Figure 3. All of them successfully yielded the desired products in very good crude purities and overall yields, which prove the robustness of the synthetic method (Table 2). We also tested 2-cyano-4-trifluorobenzenesulfonyl chloride with R^2^ = CF_3_ as the alternative electron-withdrawing group. Previously, it was shown that the CF_3_ group is not strong enough to trigger the Truce–Smiles rearrangement.^7^ Interestingly, the combination of 2-CN and 4-CF_3_ yielded the desired product **5p** (R^1^ = Me, R^2^ = CF_3_, R^3^ = Me) but in very low crude purity and isolated yield of 6% (NMR data not shown due to the low quality and impurities). This proves that the presence of a nitro group is crucial to receiving the target compounds on the preparative scale.

**Table 2:**
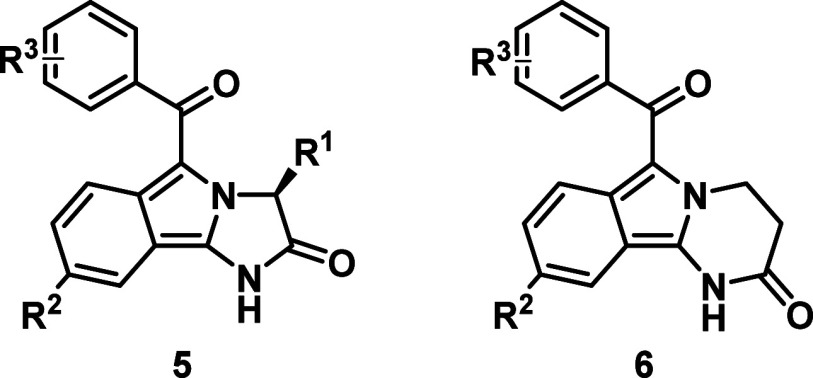
List of Synthesized and Fully Characterized Products (Using the SPS Approach)

**entry**	**cmpd.**	**R** ^**1**^	**R** ^**2**^	**R** ^**3**^	**crude purity** ^a^ **(%)**	**yield** ^b^ **(%)**
**1**	**5b**	CH_3_-	-NO_2_	4-CH_3_-	85	60
**2**	**5c**	Ph-CH_2_-	-NO_2_	4-CH_3_-	94	50
**3**	**5d**	Boc-NH-(CH_2_)_4_-	-NO_2_	4-CH_3_-	85	57
**4**	**5e**	4-*t*BuO-Ph-CH_2_-	-NO_2_	4-CH_3_-	93	44
**5**	**5f**	*t*BuOOC-(CH_2_)_2_-	-NO_2_	4-CH_3_-	98	63
**6**	**5g**	(CH_3_)_2_CH CH_2_-	-NO_2_	4-CH_3_-	79	33
**7**	**5h**	N_3_-CH_2_-CH_2_-	-NO_2_	4-CH_3_-	89	46
**8**	**5i**	*N*-Boc-indol-2-yl-CH_2_-	-NO_2_	4-CH_3_-	86	69
**9**	**5j**	Pbf-Gdm-(CH_2_)_3_-	-NO_2_	4-CH_3_-	82	80
**10**	**5k**	CH_3_-	-NO_2_	4-Cl-	88	85
**11**	**5l**	CH_3_-	-NO_2_	2-CH_3_-	93	76
**12**	**5m**	CH_3_-	-NO_2_	3,5-diCl-4-NH_2_Ph	90	88
**13**	**5n**	CH_3_-	-NO_2_	4-MeO-	91	72
**14**	**5o**	CH_3_-	-NO_2_	4-F-	95	80
**15**	**5p**	CH_3_-	-CF_3_	4-CH_3_-	39	6
**16**	**6**		-NO_2_	4-CH_3_-	96	26

a Crude purity after the entire reaction sequence calculated from UPLC-UV traces.

b Overall yield after the entire reaction sequence and final purification calculated from the loading of the resin **2**.

**Figure 3:**
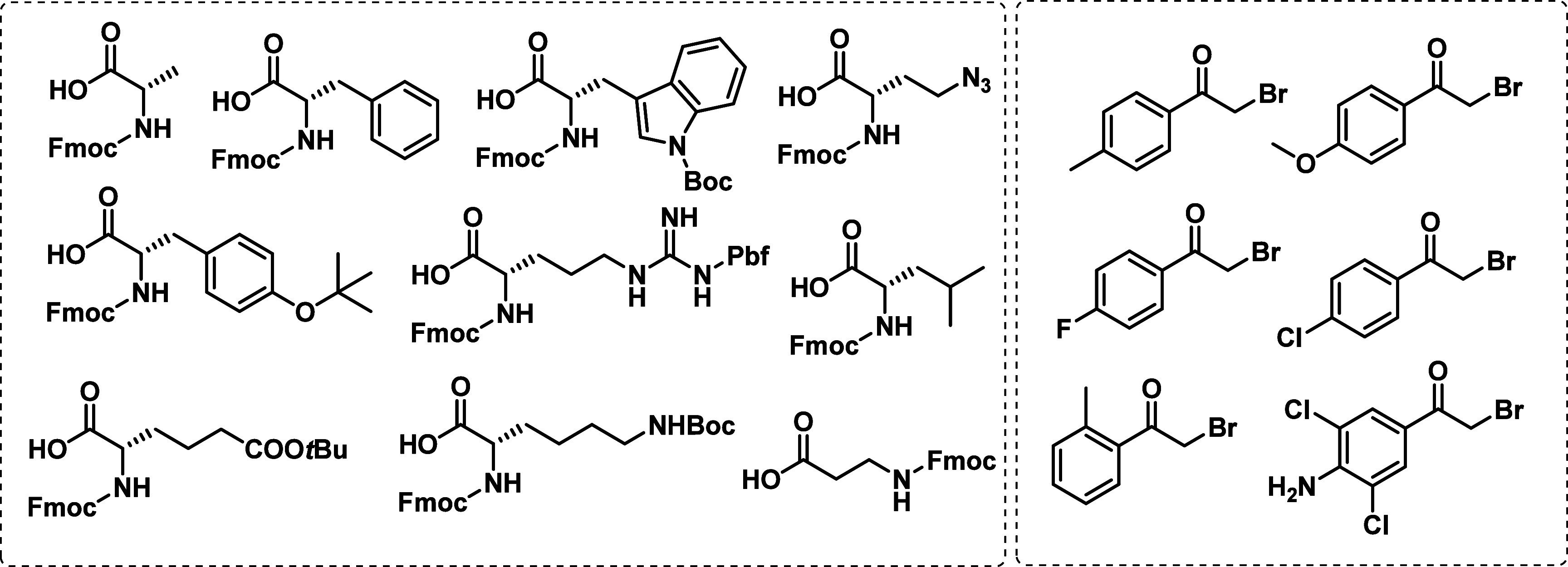
List of successfully tested building blocks.

Finally, the synthesis of target compounds *via* traditional solution-phase chemistry was verified. This alternative resembled the SPS alternative; however, methyl esters of α-amino acids were used instead of Fmoc-amino acids (Scheme 3). In contrast to polymer-supported synthesis, both reactions leading to key intermediates (i.e., sulfonylation and alkylation) were difficult to drive to completion even when an excess of reactants was used. Consequently, the corresponding products had to be purified from the starting materials by column chromatography, which influenced the yields. The final step was carried out with 2 equiv of AmAc in a mixture of ACN and water (1:1) at 100 °C for 2 h, similar to the SPS alternative. Although the cyclization provided the target compounds in very good crude purity, the yields were again compromised due to the problematic removal of impurities.

**Scheme 3:**
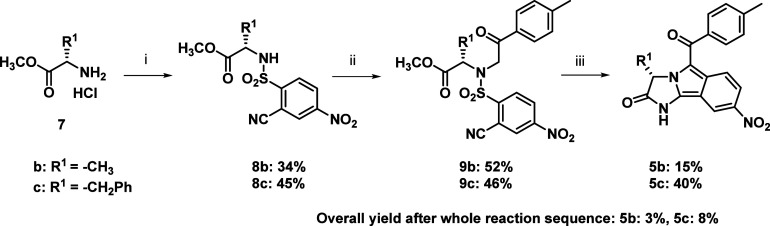
Solution-Phase Synthesis of Target Compounds^a^

a Reagents and conditions: (i) H-Ala-OMe or H-Phe-OMe, 2-cyano-4-nitrobenzenesulfonyl chloride, DIPEA, DCM, rt, 3 h; (ii) α-bromoketone, DIPEA, ACN, rt, 3 h; and (iii) ammonium acetate, ACN/water (1/1), 100 °C, 2 h.

## Conclusions

2-Cyano-4-nitrobenzenesulfonamide and 2-cyano-4-(trifluoromethyl)­benzenesulfonyl chloride can be used as the key building blocks for the preparation of novel skeletal isoindoles. The construction of the target scaffold from starting amino acids involves six reactions; however, three reactions (C-arylation, isoindole formation, and imidazo­[2,1-*a*]­isoindolones cyclization) take place in a one-step cascade fashion, which result in a three-step procedure. Amino acids and haloketones are excellent starting materials in terms of availability and can be applied in different combinations to prepare various substituted products. Additionally, the nitro group remaining in the final compounds can be considered suitable functionality for further transformation. The target imidazo­[2,1-*a*]­isoindolones are rapidly synthesized *via* conventional solid-phase synthesis and the commonly used Wang resin, with cyclative cleavage as the last step, which eliminates the need for an external cleaving agent and contributes to the crude purity of the final compounds. On the other hand, taking the limitations of solid-phase synthesis into account, the developed solution-phase chemistry protocols can be applied to prepare larger quantities of products; however, overall yields are lower because of the purification required within the reaction sequence. In general, both alternative strategies allow the simple preparation of novel isoindoles, which can be used in the search for novel bioactive molecules.

## Experimental Section

### General Information

Solvents and chemicals were purchased from Sigma-Aldrich (Milwaukee, USA, www.sigmaaldrich.com), Acros Organic (Geel, Belgium, www.acros.com), and Fluorochem (Derbyshire, UK, www.fluorochem.co.uk). The Wang resin (100–200 mesh, 1% DVB, 1.4 mmol/g) was obtained from AAPPTec (Louisville, USA, www.aapptec.com). Solid-phase synthesis was done in plastic reaction vessels (syringes, each equipped with a porous disk) using a manually operated synthesizer (Torviq, Niles, USA, www.torviq.com). Dry solvents were dried over 4 Å molecular sieves or stored as received from commercial suppliers.

All reactions were carried out at ambient temperature (23 °C) unless stated otherwise; heating was performed by using a heating metal block. For the LC/MS analyses, a sample of resin (∼5 mg) was treated with 50% TFA in DCM, the cleavage cocktail was evaporated under a stream of nitrogen, the cleaved compounds were dissolved in CH_3_CN/H_2_O (20 or 50%; 1 mL), and the resin was removed by filtration. LC/MS analyses were carried out using a UPLC-MS system consisting of a UPLC chromatograph (Acquity) with a photodiode array detector and single quadrupole mass spectrometer (Waters) using a C18 X-Select HSS T3 column (2.5 μm, 3.0 × 50 mm) at 30 °C and a flow rate of 0.6 mL/min. The mobile phase was (A) 0.01 M ammonium acetate (AmAc) in H_2_O and (B) CH_3_CN with linearly programmed gradient elution. The ESI source was operated at a capillary voltage of 3 kV, desolvation temperature of 350 °C, and source temperature of 120 °C. The purification was performed by the normal phase (silica gel chromatography) or using semipreparative HPLC (Waters, USA) equipped with a PDA and MS detector. All separations were done using a C18 reverse phase column (YMC-Actus Pro C18 column, 20 × 100 mm, 5 μm particle size). The mobile phase consisted of an aqueous solution of 0.01 M ammonium acetate and HPLC gradient grade acetonitrile. A linear gradient was formed by acetonitrile in aqueous ammonium acetate for 6 min with a flow rate of 15 mL/min. The precise purification method is specified for each product in the experimental procedures. Residual solvents (H_2_O and AmAc buffer) were lyophilized by the ScanVac Coolsafe 110-4 operating at −110 °C. HRMS analysis was performed using LC-MS (Dionex Ultimate 3000, Thermo Fischer Scientific, USA) with an Exactive Plus Orbitrap high-resolution mass spectrometer (Thermo Exactive plus, Thermo Fischer Scientific, USA) operating at positive or negative full scan mode (120,000 FWMH) in the range of 100–1000 *m*/*z* with electrospray ionization operating at 150 °C and the source voltage of 3.6 kV. Chromatographic separation was performed on a Phenomenex Gemini column (C18, 50 × 2 mm, 3 μm particle size) with isocratic elution and mobile phase (MP) containing MeOH/H_2_O/formic acid 95:5:0.1. The samples were dissolved in CH_3_CN or MeOH/H_2_O (95:5 v/v). NMR experiments were performed with the use of the ECA400II spectrometer (JEOL) at magnetic field strengths of 9.39 T with operating frequencies of 399.78 MHz for ^1^H and 100.53 MHz for ^13^C at a temperature 27 °C Chemical shifts (δ) are reported in parts per million (ppm), and coupling constants (*J*) are reported in hertz (Hz). The signals of DMSO-*d*_6_ and CDCl_3_ were set at 2.50 and 7.26 ppm in ^1^H NMR spectra and at 39.50 and 77.00 ppm in ^13^C NMR spectra. Abbreviations in NMR spectra are as follows: br. s, broad singlet; s, singlet; d, doublet; dd, doublet of doublets; ddd, doublet of doublets of doublets; dt, doublet of triplets; t, triplet; and m, multiplet.

#### General Method for Calculation of Yields Using ^1^H NMR

^1^H NMR spectra of an external standard in DMSO-*d*_6_ at three different concentrations were measured. In each spectrum, the solvent signal was integrated followed by the integration of selected H_Ar_ signals of external standard. Ratios of solvent/standard signal areas along with a known quantity of the standard were used to construct a calibration curve. Then, the ^1^H NMR spectra of the studied sample were measured, and the ratio of solvent/sample (selected H_Ar_ signal) areas was determined. Using the calibration curve, the quantity of compound in the sample was calculated.

#### Quantification of the Resin Loading

Quantification of the loading of the Wang resin with immobilized Fmoc-AA–OH was carried out as follows: After immobilization, the sample of resin (∼30 mg) was washed with DCM (3×) and MeOH (5×), dried, and divided into two samples (2 × 10 mg). Both samples were cleaved from the resin using TFA in DCM (0.5 mL, 50%) for 2 h at ambient temperature. The cleavage cocktail was evaporated by a stream of nitrogen, and the oily residue was extracted into 1 mL of MeOH and analyzed by UPLC-UV-MS. The loading of resin was calculated with the use of an external standard (Fmoc-Ala-OH, 0.5 mg/mL).

#### Solid-Phase Synthesis Approach

##### Immobilization of Fmoc-AA-OH on the Wang Resin (**2**)

The Wang resin (1 g, 1.4 mmol/g) was washed with DCM (3×) and DMF (3×). A solution of Fmoc-AA-OH (2 mmol), HOBt (306 mg, 2.0 mmol), DMAP (61 mg, 0.5 mmol), and DIC (312 μL, 2.0 mmol) in DMF/DCM (10 mL, 50%) was added to the swollen resin, and the reaction mixtures were shaken for 24 h at ambient temperature. After the reaction, the resin was washed with DMF (3×) and DCM (3×). The quantification was carried out as described in the “Quantification of the Resin Loading” section.

##### Nosylation with 2-Cyano-4-nitrobenzenesulfonyl Chloride (**3**)

Resin **2** (250 mg) was treated with 20% piperidine in DMF (2.5 mL) for 20 min and subsequently washed with DMF (3×) and DCM (5×). A solution of 2-cyano-4-nitrobenzenesulfonyl chloride (74 mg, 0.3 mmol) and 2,6-lutidine (52 μL, 0.45 mmol) in DCM (2 mL) was added to the resin, and the resin slurry was shaken for 16 h. Resins were then washed with DCM (5×) and analyzed using the standard procedure.

##### Alkylation with α-Bromoketones (**4**)

Resin **3** (240 mg) was washed with DMF (3×), and a 0.5 M solution of α-bromoketone (1.25 mmol) and DIPEA (217 μL, 1.25 mmol) in DMF (2.5 mL) was added. The reaction mixtures were shaken for 4 h at ambient temperature. The resin was washed with DMF (3×) and DCM (3×) and analyzed using the standard procedure.

##### Cyclization to Fused Isoindoles (**5**)

Resin **4** (230 mg) was washed with ACN (3×) and transferred to glass reaction vessels using 2 mL of ACN. Subsequently, ammonium acetate (30.8 mg, 0.4 mmol) was added followed by the addition of 2 mL of water. The resulting reaction mixture was heated at 100 °C for 2 h. After the reaction, the glass vessel was cooled, and the resin was filtered and washed with ACN (3×) and DCM (3×). The collected volumes were collected, evaporated, and purified by column chromatography using hexane/ethyl acetate (1/1) for **5b–c**, **5e–f**, and **5i**; ethyl acetate for **5d** and **5k**; and CH_2_Cl_2_/methanol (95/5) for **5h**, **5j**, and **6**.

##### (*S*)-3-Methyl-5-(4-methylbenzoyl)-8-nitro-1*H*-imidazo­[2,1-*a*]­isoindol-2­(3*H*)-one (**5b**)

Reddish brown solid, 28.8 mg (yield 60%); *R*_f_ = 0.42 (SiO_2_, hexane/EA = 1/1). ^1^H NMR (400 MHz, DMSO-*d*_6_) δ 12.40 (s, 1H), 8.69 (dd, *J* = 2.2, 0.5 Hz, 1H), 7.79 (dd, *J* = 9.5, 2.2 Hz, 1H), 7.56 (dt, *J* = 8.1, 1.8 Hz, 2H), 7.37–7.39 (m, 2H), 6.97 (dd, *J* = 9.5, 0.5 Hz, 1H), 5.27 (q, *J* = 7.0 Hz, 1H), 2.44 (s, 3H), 1.68 (d, *J* = 7.2 Hz, 3H) ppm. ^13^C­{^1^H} NMR (101 MHz, DMSO-*d*_6_) δ: 181.0, 175.9, 141.4, 141.3, 140.6, 137.3, 130.7, 129.1, 128.2, 120.1, 119.9, 119.6, 115.2, 105.8, 59.8, 21.1, 16.0 ppm. HRMS (ESI): *m*/*z* calcd C_19_H_15_N_3_O_4_ for [M + H]^+^ = 350.1135, found [M + H]^+^ = 350.1130.

##### (*S*)-3-Benzyl-5-(4-methylbenzoyl)-8-nitro-1*H*-imidazo­[2,1-*a*]­isoindol-2­(3*H*)-one (**5c**)

Dark orange solid, 25.1 mg (yield 50%); *R*_f_ = 0.63 (SiO_2_, hexane/EA = 1/1). ^1^H NMR (400 MHz, chloroform-*d*) δ 9.71 (s, 1H), 8.44 (d, *J* = 2.0 Hz, 1H), 7.76 (dd, *J* = 9.5, 2.1 Hz, 1H), 7.69 (d, *J* = 7.9 Hz, 2H), 7.37 (d, *J* = 7.9 Hz, 2H), 7.10–7.04 (m, 4H), 6.95–6.93 (m, 2H), 5.71 (q, *J* = 2.8 Hz, 1H), 3.94 (dd, *J* = 14.2, 5.6 Hz, 1H), 3.61 (dd, *J* = 14.2, 2.9 Hz, 1H), 2.51 (s, 3H) ppm. ^13^C­{^1^H} NMR (101 MHz, chloroform-*d*) δ: 182.9, 174.8, 142.6, 141.9, 140.1, 136.9, 133.4, 131.8, 131.3, 129.5, 129.3, 128.7, 128.4, 127.5, 120.9, 120.5, 118.9, 106.0, 65.0, 35.6, 21.7 ppm. HRMS (ESI): *m*/*z* calcd C_25_H_20_N_3_O_4_ for [M + H]^+^ = 426.1448, found [M + H]^+^ = 426.1442.

##### *tert*-Butyl (*S*)-(4-(5-(4-Methylbenzoyl)-8-nitro-2-oxo-2,3-dihydro-1*H*-imidazo­[2,1-*a*]­isoindol-3-yl)­butyl)­carbamate (**5d**)

Redish brown solid, 34 mg (yield 57%); *R*_f_ = 0.32 (SiO_2_, hexane/EA = 1/1). ^1^H NMR (400 MHz, DMSO-*d*_6_) δ 12.47 (s, 1H), 8.70 (dd, *J* = 2.3, 0.6 Hz, 1H), 7.80 (dd, *J* = 9.6, 2.3 Hz, 1H), 7.57 (d, *J* = 7.9 Hz, 2H), 7.38 (d, *J* = 7.8 Hz, 2H), 6.98 (dd, *J* = 9.5, 0.5 Hz, 1H), 6.65 (t, *J* = 5.3 Hz, 1H), 5.33 (dd, *J* = 6.3, 2.8 Hz, 1H), 2.77 (dd, *J* = 13.0, 6.2 Hz, 2H), 2.44 (s, 3H), 2.26–2.34 (m, 1H), 2.04–2.13 (m, 1H), 1.31–1.37 (m, 2H), 1.27 (s, 9H), 1.01–1.10 (m, 1H), 0.84–0.93 (m, 1H) ppm. ^13^C­{^1^H} NMR (101 MHz, DMSO-*d*_6_) δ: 181.3, 175.2, 155.5, 141.6, 141.4, 140.7, 137.3, 130.8, 129.2, 128.2, 120.2, 119.9, 119.6, 115.4, 105.6, 77.2, 63.7, 29.1, 29.1, 28.1, 21.1, 20.2 ppm. HRMS (ESI): *m*/*z* calcd C_27_H_30_N_4_O_6_ for [M + H]^+^ = 507.2238, found [M + H]^+^ = 507.2246.

##### (*S*)-3-(4-(*tert*-Butoxy)­benzyl)-5-(4-methylbenzoyl)-8-nitro-1*H*-imidazo­[2,1-*a*]­isoindol-2­(3*H*)-one (**5e**)

Dark orange solid, 22.4 mg (yield 44%); *R*_f_ = 0.65 (SiO_2_, hexane/EA = 1/1). ^1^H NMR (400 MHz, chloroform-*d*) δ 10.63 (s, 1H), 7.86 (d, *J* = 1.5 Hz, 1H), 7.78 (d, *J* = 8.1 Hz, 2H), 7.57 (dd, *J* = 9.6, 2.0 Hz, 1H), 7.46 (d, *J* = 7.8 Hz, 2H), 7.01–6.98 (m, 3H), 6.90 (d, *J* = 8.5 Hz, 2H), 5.64 (dd, *J* = 5.6, 2.3 Hz, 1H), 3.94 (dd, *J* = 14.0, 5.6 Hz, 1H), 3.60 (dd, *J* = 14.0, 2.3 Hz, 1H), 2.53 (s, 3H), 1.30 (s, 9H) ppm. ^13^C­{^1^H} NMR (101 MHz, chloroform-*d*) δ: 182.8, 174.7, 153.0, 142.6, 141.5, 139.7, 136.8, 131.1, 130.3, 129.6, 128.9, 124.3, 120.8, 120.1, 117.8, 117.7, 116.7, 105.3, 80.9, 65.3, 34.9, 28.6, 21.7 ppm. HRMS (ESI): *m*/*z* calcd C_29_H_28_N_3_O_5_ for [M + H]^+^ = 498.2029, found [M + H]^+^ = 498.2037.

##### *tert*-Butyl (*S*)-3-(5-(4-Methylbenzoyl)-8-nitro-2-oxo-2,3-dihydro-1*H*-imidazo­[2,1-*a*]­isoindol-3-yl)­propanoate (**5f**)

Red solid, 23.6 mg (yield 63%); *R*_f_ = 0.61 (SiO_2_, hexane/EA = 1/1). ^1^H NMR (400 MHz, chloroform-*d*) δ 10.35 (s, 1H), 8.57 (d, *J* = 1.8 Hz, 1H), 7.75 (dd, *J* = 9.6, 2.1 Hz, 1H), 7.66 (d, *J* = 8.1 Hz, 2H), 7.33 (d, *J* = 7.9 Hz, 2H), 7.08 (d, *J* = 9.6 Hz, 1H), 5.48–5.46 (m, 1H), 2.77–2.71 (m, 2H), 2.48 (s, 3H), 2.30 (t, *J* = 7.7 Hz, 2H), 1.37 (s, 9H) ppm. ^13^C­{^1^H} NMR (101 MHz, chloroform-*d*) δ: 183.1, 174.8, 171.5, 142.5, 142.0, 139.6, 136.8, 131.7, 129.4, 128.7, 121.1, 120.4, 118.8, 116.4, 106.1, 81.5, 62.8, 29.9, 28.0, 25.9, 21.7 ppm. HRMS (ESI): *m*/*z* calcd C_25_H_25_N_3_O_6_ for [M + H]^+^ = 464.1822, found [M + H]^+^ = 464.1827.

##### (*S*)-3-Isobutyl-5-(4-methylbenzoyl)-8-nitro-1*H*-imidazo­[2,1-*a*]­isoindol-2­(3*H*)-one (**5g**)

Dark orange solid, 22.7 mg (yield 33%); *R*_f_ = 0.39 (SiO_2_, hexane/EA = 2/1). ^1^H NMR (400 MHz, chloroform-*d*) δ 10.70 (s, 1H), 8.66 (d, *J* = 2.0 Hz, 1H), 7.78 (dd, *J* = 9.6, 2.1 Hz, 1H), 7.66 (d, *J* = 8.1 Hz, 2H), 7.32 (d, *J* = 7.9 Hz, 2H), 7.07 (d, *J* = 9.6 Hz, 1H), 5.47 (t, *J* = 5.8 Hz, 1H), 2.47 (s, 3H), 2.26–2.22 (m, 2H), 1.94–1.84 (m, 1H), 0.98 (d, *J* = 6.6 Hz, 3H), 0.75 (d, *J* = 6.7 Hz, 3H) ppm. ^13^C­{^1^H} NMR (101 MHz, chloroform-*d*) δ: 183.0, 175.6, 142.5, 142.0, 139.8, 136.9, 132.0, 129.4, 128.7, 121.0, 120.4, 119.0, 116.3, 106.3, 62.9, 39.1, 24.0, 23.1, 22.1, 21.7 ppm. HRMS (ESI): *m*/*z* calcd C_22_H_21_N_3_O_4_ for [M + H]^+^ = 392.1610, found [M + H]^+^ = 392.1608.

##### (*S*)-3-(2-Azidoethyl)-5-(4-methylbenzoyl)-8-nitro-1*H*-imidazo­[2,1-*a*]­isoindol-2­(3*H*)-one (**5h**)

Dark orange solid, 14.8 mg (yield 46%); *R*_f_ = 0.41 (SiO_2_, DCM/MeOH = 95/5). ^1^H NMR (400 MHz, DMSO-*d*_6_) δ 12.55 (s, 1H), 8.69 (d, *J* = 2.0 Hz, 1H), 7.80 (dd, *J* = 9.6, 2.1 Hz, 1H), 7.58 (d, *J* = 7.9 Hz, 2H), 7.38 (d, *J* = 7.9 Hz, 2H), 6.96 (d, *J* = 9.6 Hz, 1H), 5.41 (dd, *J* = 7.2, 2.8 Hz, 1H), 3.49–3.43 (m, 1H), 3.22–3.15 (m, 1H), 2.69–2.60 (m, 1H), 2.48–2.44 (m, 4H) ppm. ^13^C­{^1^H} NMR (101 MHz, DMSO-*d*_6_) δ: 181.2, 174.9, 142.1, 141.4, 140.8, 137.3, 130.8, 129.2, 128.2, 120.2, 120.1, 119.6, 115.6, 105.8, 61.6, 46.0, 28.5, 21.1 ppm. HRMS (ESI): *m*/*z* calcd C_20_H_16_N_6_O_4_ for [M + H]^+^ = 405.1311, found [M + H]^+^ = 405.1310.

##### *tert*-Butyl (*S*)-3-((5-(4-Methylbenzoyl)-8-nitro-2-oxo-2,3-dihydro-1 *H* -imidazo­[2,1- *a* ]­isoindol-3-yl)­methyl)-1*H*-indole-1-carboxylate (**5i**)

Dark orange solid, 31.4 mg (yield 69%); *R*_f_ = 0.69 (SiO_2_, Hexane/EA = 1/1). ^1^H NMR (400 MHz, chloroform-*d*) δ 9.63 (s, 1H), 8.39 (d, *J* = 1.8 Hz, 1H), 7.94 (d, *J* = 8.1 Hz, 1H), 7.74 (dd, *J* = 9.6, 2.1 Hz, 1H), 7.67 (d, *J* = 7.9 Hz, 2H), 7.48 (d, *J* = 7.9 Hz, 1H), 7.35 (d, *J* = 7.9 Hz, 2H), 7.16 (t, *J* = 7.3 Hz, 1H), 7.09–7.02 (m, 3H), 5.69 (q, *J* = 2.7 Hz, 1H), 4.04 (dd, *J* = 15.1, 5.6 Hz, 1H), 3.75 (dd, *J* = 15.1, 2.7 Hz, 1H), 2.51 (s, 3H), 1.49 (s, 9H) ppm. ^13^C­{^1^H} NMR (101 MHz, chloroform-*d*) δ: 183.0, 174.7, 149.2, 142.5, 142.0, 139.8, 137.0, 135.0, 131.6, 130.0, 129.4, 128.7, 124.7, 124.5, 122.6, 121.0, 120.4, 119.1, 118.8, 116.5, 114.9, 112.5, 106.0, 83.8, 64.3, 28.0, 25.4, 21.7 ppm. HRMS (ESI): *m*/*z* calcd C_32_H_28_N_4_O_6_ for [M + H]^+^ = 565.2087, found [M + H]^+^ = 565.2057.

##### (*S*)-2,2,4,6,7-Pentamethyl-*N*-(*N*-(3-(5-(4-methylbenzoyl)-8-nitro-2-oxo-2,3-dihydro-1*H*-imidazo­[2,1-*a*]­isoindol-3-yl)­propyl)­carbamimidoyl)-2,3-dihydrobenzofuran-5-sulfonamide (**5j**)

Orange solid, 49.6 mg (yield 85%); *R*_f_ = 0.40 (SiO_2_, DCM/MeOH = 95/5). ^1^H NMR (400 MHz, chloroform-*d*) δ 11.67 (s, 1H), 8.67 (s, 1H), 7.59–7.55 (m, 3H), 7.25 (d, *J* = 8.4 Hz, 2H, overlap with CHCl_3_), 6.87 (d, *J* = 9.5 Hz, 1H), 6.39 (s, 2H), 5.34 (s, 1H), 3.21 (s, 2H), 2.86 (s, 2H), 2.44–2.39 (m, 12H), 1.97 (s, 3H), 1.60–1.41 (m, 8H) ppm. ^13^C­{^1^H} NMR (101 MHz, chloroform-*d*) δ: 182.6, 175.6, 168.0, 158.9, 142.3, 141.7, 141.1, 138.5, 136.8, 132.3, 129.3, 128.6, 124.8, 120.5, 120.4, 120.4, 119.7, 119.6, 117.6, 115.8, 106.7, 86.6, 63.2, 43.0, 40.5, 28.5, 27.8, 23.4, 21.6, 19.2, 17.8, 12.3 ppm. HRMS (ESI): *m*/*z* calcd C_35_H_38_N_6_O_7_S for [M + H]^+^ = 687.2601, found [M + H]^+^ = 687.2613.

##### (*S*)-5-(4-Chlorobenzoyl)-3-methyl-8-nitro-1*H*-imidazo­[2,1-*a*]­isoindol-2­(3*H*)-one (**5k**)

Dark orange solid, 39 mg (yield 85%); *R*_f_ = 0.81 (SiO_2_, EA). ^1^H NMR (400 MHz, DMSO-*d*_6_) δ 12.47 (s, 1H), 8.68 (dd, *J* = 2.3, 0.6 Hz, 1H), 7.83 (dd, *J* = 9.5, 2.2 Hz, 1H), 7.62–7.68 (m, 4H), 6.92 (dd, *J* = 9.5, 0.4 Hz, 1H), 5.26 (q, *J* = 7.1 Hz, 1H), 1.70 (d, *J* = 7.2 Hz, 3H) ppm. ^13^C­{^1^H} NMR (101 MHz, DMSO-*d*_6_) δ: 179.6, 175.9, 142.1, 140.8, 138.9, 135.9, 131.2, 129.9, 128.8, 120.4, 120.0, 119.6, 115.0, 106.2, 59.9, 16.0 ppm. HRMS (ESI): *m*/*z* calcd C_18_H_12_ClN_3_O_4_ for [M + H]^+^ = 370.0589, found [M + H]^+^ = 370.0591.

##### (*S*)-3-Methyl-5-(2-methylbenzoyl)-8-nitro-1*H*-imidazo­[2,1-*a*]­isoindol-2­(3*H*)-one (**5l**)

Redish brown solid, 33 mg (yield 76%); *R*_f_ = 0.74 (SiO_2_, EA). ^1^H NMR (400 MHz, chloroform-*d*) δ 8.66 (dd, *J* = 2.1, 0.5 Hz, 1H), 7.75 (dd, *J* = 9.5, 1.9 Hz, 1H), 7.41–7.47 (m, 1H), 7.28–7.35 (m, 3H), 6.41 (d, *J* = 10.1 Hz, 1H), 5.38–5.43 (m, 1H), 2.30 (s, 3H), 1.99 (d, *J* = 8.1 Hz, 3H) ppm. ^13^C­{^1^H} NMR (101 MHz, DMSO-*d*_6_) δ: 181.5, 176.1, 142.3, 140.8, 140.5, 134.4, 131.6, 130.7, 129.6, 126.7, 126.1, 120.4, 119.7, 119.2, 115.7, 106.3, 60.0, 18.7, 16.0 ppm. HRMS (ESI): *m*/*z* calcd C_19_H_15_N_3_O_4_ for [M + H]^+^ = 350.1135, found [M + H]^+^ = 350.1138.

##### (*S*)-5-(4-Amino-3,5-dichlorobenzoyl)-3-methyl-8-nitro-1*H*-imidazo­[2,1-*a*]­isoindol-2­(3*H*)-one (**5m**)

Reddish brown solid, 48.7 mg (yield 88%); *R*_f_ = 0.8 (SiO_2_, EA). ^1^H NMR (400 MHz, DMSO-*d*_6_) δ 12.37 (s, 1H), 8.68 (d, *J* = 2.1 Hz, 1H), 7.85 (dd, *J* = 9.5, 2.2 Hz, 1H), 7.55 (s, 2H), 7.21 (d, *J* = 9.6 Hz, 1H), 6.28 (s, 2H), 5.23 (q, *J* = 7.1 Hz, 1H), 1.63 (d, *J* = 7.2 Hz, 3H) ppm. ^13^C­{^1^H} NMR (101 MHz, DMSO-*d*_6_) δ: 177.8, 176.2, 144.3, 141.6, 140.5, 130.3, 128.7, 127.6, 120.0, 119.9, 119.7, 117.4, 114.7, 105.7, 59.6, 15.9 ppm. HRMS (ESI): *m*/*z* calcd C_18_H_12_Cl_2_N_4_O_4_ for [M + H]^+^ = 419.0308, found [M + H]^+^ = 419.0314.

##### (*S*)-5-(4-Methoxybenzoyl)-3-methyl-8-nitro-1*H*-imidazo­[2,1-*a*]­isoindol-2­(3*H*)-one (**5n**)

Dark orange solid, 32.8 mg (yield 72%); *R*_f_ = 0.68 (SiO_2_, EA). ^1^H NMR (400 MHz, DMSO-*d*_6_) δ 12.38 (s, 1H), 8.68 (dd, *J* = 2.3, 0.5 Hz, 1H), 7.79 (dd, *J* = 10.0, 2.5 Hz, 1H), 7.64–7.67 (m, 2H), 7.09–7.12 (m, 2H), 7.07 (dd, *J* = 9.5, 0.4 Hz, 1H), 5.27 (q, *J* = 7.1 Hz, 1H), 3.88 (s, 3H), 1.66 (d, *J* = 7.2 Hz, 3H) ppm. 13C­{^1^H} NMR (101 MHz, DMSO-*d*_6_) δ 180.5, 175.9, 161.9, 141.1, 140.6, 132.2, 130.4, 130.3, 120.2, 119.7, 119.7, 115.2, 113.9, 105.6, 59.8, 55.4, 16.0 ppm. HRMS (ESI): *m*/*z* calcd C_19_H_15_N_3_O_5_ for [M + H]^+^ = 366.1084, found [M + H]^+^ = 366.1086.

##### (*S*)-5-(4-Fluorobenzoyl)-3-methyl-8-nitro-1*H*-imidazo­[2,1-*a*]­isoindol-2­(3*H*)-one (**5o**)

Reddish brown solid, 32 mg (yield 80%); *R*_f_ = 0.82 (SiO_2_, EA). ^1^H NMR (400 MHz, DMSO-*d*_6_) δ 12.44 (s, 1H), 8.66 (s, 1H), 7.80 (dd, *J* = 9.5, 1.7 Hz, 1H), 7.69–7.73 (m, 2H), 7.38–7.42 (m, 2H), 6.91 (d, *J* = 9.6 Hz, 1H), 5.26 (q, *J* = 7.1 Hz, 1H), 1.69 (d, *J* = 7.2 Hz, 3H) ppm. ^13^C­{^1^H} NMR (101 MHz, DMSO-*d*_*6*_) δ 179.7, 175.9, 163.8 (d, ^1^ *J*_C–F_ = 248.7 Hz, 1C), 141.8, 140.7, 136.6 (d, ^4^ *J*_C–F_ = 2.9 Hz, 1C), 131.0, 130.7 (d, ^3^ *J*_C–F_ = 8.6 Hz, 1C), 120.2, 119.9, 119.5, 115.7 (d, ^2^ *J*_C–F_ = 22.0 Hz, 1C), 115.0, 106.0, 59.9, 15.9 ppm. HRMS (ESI): *m*/*z* calcd C_18_H_12_FN_3_O_4_ for [M + H]^+^ = 354.0885, found [M + H]^+^ = 354.0885.

##### 6-(4-Methylbenzoyl)-9-nitro-3,4-dihydropyrimido­[2,1-*a*]­isoindol-2­(1*H*)-one (**6**)

Yellow solid, 12.6 mg (yield 26%); *R*_f_ = 0.47 (SiO_2_, DCM/MeOH = 95/5). ^1^H NMR (400 MHz, DMSO-*d*_6_) δ 12.07 (s, 1H), 9.08 (dd, *J* = 2.1, 0.5 Hz, 1H), 7.71 (dd, *J* = 9.5, 2.2 Hz, 1H), 7.55–7.53 (m, 2H), 7.37–7.35 (m, 2H), 6.69 (dd, *J* = 9.5, 0.5 Hz, 1H), 4.93 (t, *J* = 7.2 Hz, 2H), 2.95 (t, *J* = 7.2 Hz, 2H), 2.43 (s, 3H) ppm. ^13^C­{^1^H} NMR (101 MHz, DMSO-*d*_6_) δ: 182.3, 167.0, 141.4, 140.5, 138.0, 136.4, 129.6, 129.1, 128.5, 119.7, 119.7, 119.4, 115.3, 109.3, 41.9, 29.7, 21.1 ppm. HRMS (ESI): *m*/*z* calcd C_19_H_15_N_3_O_4_ for [M + H]^+^ = 350.1141, found [M + H]^+^ = 350.1136.

#### Procedures for the Solution-Phase Approach

##### Preparation of Sulfonamides (**8b**, **8c**)

To a stirred solution of l-amino acids methyl ester hydrochloride (0.8 mmol) and 2-cyano-4-nitrobenzenesulfonyl chloride (237 mg; 0.96 mmol) in 4 mL of dichloromethane (DCM), diisopropylethylamine (342 μL, 1.92 mmol) was added, and the reaction mixture was stirred for 3 h. Then water (15 mL) was added, and the product was extracted with DCM (3× 15 mL). The combined organic layers were dried using MgSO_4_, filtered, and evaporated to dryness *in vacuo*. The resulting crude products were purified by column chromatography using 2% MeOH in DCM.

##### Methyl ((2-Cyano-4-nitrophenyl)­sulfonyl)-l-alaninate (**8b**)

White solid, 103 mg (yield 34%); *R*_f_ = 0.49 (SiO_2_, 2% MeOH in DCM). The product was obtained as a mixture of two rotamers in a 10:6 ratio. In the proton spectrum, peaks belonging to a major rotamer are designated as M, and peaks belonging to a minor rotamer are designated as m. Signals in the carbon spectrum were not assigned. ^1^H NMR (400 MHz, DMSO-*d*_6_) δ 10.34 (s, 1H, m), 9.37 (d, *J* = 1.8 Hz, 1H, m), 9.16 (s, 1H, M), 8.91 (d, *J* = 2.3 Hz, 1H, M), 8.68–8.65 (m, 2H, both rotamers), 8.49 (d, *J* = 8.5 Hz, 1H, m), 8.25 (d, *J* = 8.7 Hz, 1H, M), 5.10 (q, *J* = 7.2 Hz, 1H, m), 4.18 (q, *J* = 7.2 Hz, 1H, M), 3.63 (s, 3H, m), 3.49 (s, 3H, M), 1.67 (d, *J* = 7.2 Hz, 3H, m), 1.32 (d, *J* = 7.2 Hz, 3H, M) ppm. ^13^C­{^1^H} NMR (101 MHz, DMSO-*d*_6_) δ: 171.8, 169.4, 151.5, 149.2, 149.1, 148.0, 138.5, 130.6, 130.5, 128.8, 128.6, 127.0, 123.1, 120.4, 114.4, 110.5, 52.4, 52.1, 51.5, 48.9, 18.1, 14.3 ppm. HRMS (ESI): *m*/*z* calcd C_11_H_11_N_3_O_6_S for [M + H]^+^ = 314.0441, found [M + H]^+^ = 314.0449.

##### Methyl ((2-Cyano-4-nitrophenyl)­sulfonyl)-l-phenylalaninate (**8c**)

White solid, 140 mg (yield 45%); *R*_f_ = 0.65 (SiO_2_, 2% MeOH in DCM). ^1^H NMR (400 MHz, chloroform-*d*) δ 8.44 (d, *J* = 2.3 Hz, 1H), 8.38 (dd, *J* = 8.5, 2.4 Hz, 1H), 8.07 (d, *J* = 8.7 Hz, 1H), 7.11–7.18 (m, 3H), 7.04–7.07 (m, 2H), 5.69 (s, 1H), 4.39–4.42 (m, 1H), 3.73 (s, 3H), 3.19 (dd, *J* = 14.2, 4.9 Hz, 1H), 2.96 (dd, *J* = 14.0, 8.7 Hz, 1H) ppm. ^13^C­{^1^H} NMR (101 MHz, chloroform-*d*) δ: 170.8, 149.1, 148.1, 134.8, 130.3, 129.6, 129.2, 128.8, 127.3, 127.3, 114.2, 112.0, 57.9, 53.0, 38.8 ppm. HRMS (ESI): *m*/*z* calcd C_17_H_15_N_3_O_6_S for [M + H]^+^ = 390.0754, found [M + H]^+^ = 390.0757.

##### Alkylation with α-Bromoketone (**9b**, **9c**)

The starting sulfonamide (1 equiv) was dissolved in ACN (300 μL per 0.13 mmol) followed by addition of 3 equiv of 2-bromo-1-(4-methylphenyl)­ethan-1-one and 1.2 equiv of DIPEA (27 μL, 0.16 mmol). The reaction mixture was stirred at room temperature for 3 h. ACN was evaporated, and the crude product was purified by column chromatography using hexane/ethyl acetate (2/1) as the mobile phase.

##### Methyl *N*-((2-Cyano-4-nitrophenyl)­sulfonyl)-*N*-(2-oxo-2-(p-tolyl)­ethyl)-l-alaninate (**9b**)

Light orange solid, 64 mg (yield 52%); *R*_f_ = 0.35 (SiO_2_, hexane/EA = 2/1). ^1^H NMR (400 MHz, chloroform-*d*) δ 8.68 (d, *J* = 2.0 Hz, 1H), 8.48 (dd, *J* = 8.7, 2.4 Hz, 1H), 8.27 (d, *J* = 8.7 Hz, 1H), 7.75 (dt, *J* = 8.5, 1.9 Hz, 2H), 7.25–7.28 (m, 2H), 5.22 (d, *J* = 19.1 Hz, 1H), 4.98–5.04 (m, 1H), 4.84 (d, *J* = 19.2 Hz, 1H), 3.68 (s, 3H), 2.41 (s, 3H), 1.44 (d, *J* = 7.3 Hz, 3H) ppm. ^13^C­{^1^H} NMR (101 MHz, chloroform-*d*) δ: 193.0, 171.4, 149.1, 148.7, 145.3, 131.5, 129.8, 129.6, 128.0, 127.2, 115.0, 112.7, 56.4, 52.7, 51.7, 21.7, 16.3 ppm. HRMS (ESI): *m*/*z* calcd C_20_H_19_N_3_O_7_S for [M + H]^+^ = 446.1016, found [M + H]^+^ = 446.1016.

##### Methyl *N*-((2-Cyano-4-nitrophenyl)­sulfonyl)-*N*-(2-oxo-2-(*p*-tolyl)­ethyl)-l-phenylalaninate (**9c**)

Light orange solid, 31.5 mg (yield 46%); *R*_f_ = 0.52 (SiO_2_, hexane/EA = 2/1). ^1^H NMR (400 MHz, chloroform-*d*) δ 8.52 (d, *J* = 2.3 Hz, 1H), 8.43 (dd, *J* = 8.7, 2.3 Hz, 1H), 8.24 (d, *J* = 8.9 Hz, 1H), 7.79 (dt, *J* = 8.3, 1.8 Hz, 2H), 7.28–7.31 (m, 2H), 7.19–7.23 (m, 3H), 7.13–7.17 (m, 2H), 5.23 (d, *J* = 18.9 Hz, 1H), 5.16 (d, *J* = 18.8 Hz, 1H), 4.89 (dd, *J* = 8.5, 6.7 Hz, 1H), 3.51 (s, 3H), 3.03–3.16 (m, 2H), 2.43 (s, 3H) ppm. ^13^C­{^1^H} NMR (101 MHz, chloroform-*d*) δ: 193.0, 169.5, 149.1, 148.3, 145.2, 135.2, 131.8, 131.6, 129.7, 129.6, 129.4, 128.7, 128.1, 127.2, 127.0, 115.0, 112.6, 61.3, 52.5, 51.8, 36.7, 21.8 ppm. HRMS (ESI): *m*/*z* calcd C_26_H_23_N_3_O_7_S for [M + H]^+^ = 522.1329, found [M + H]^+^ = 522.1323.

##### Cyclization to Fused Isoindoles (**5b**, **5c**)

The corresponding intermediate **9b** or **9c** (1 equiv) was dissolved in 2 mL of ACN with subsequent addition of 2 mL of ammonium acetate solution in water prepared by dissolving 2 equiv of ammonium acetate. The reaction mixture was stirred at 100 °C for 2 h. The solution was cooled, and the crude product was purified by semipreparative RP-HPLC. NMR and HRMS spectra of **5b** and **5c** correspond with the results obtained by the SPS approach.

## Supplementary Material



## Data Availability

The data underlying this study are available in the published article and in its Supporting Information and are openly available in the Palacký University cloud storage at https://upolomouc.sharepoint.com/:f:/s/msresearchgroup/Ei_-GouTOMlGu7ZSlfT0VaQB14Mbgs0VenQNu-cVJTbWSQ?e=fQZnww.

## Notes

NMR spectra of synthesized compounds (PDF)

## Notes

This work is dedicated to Viktor Krchňák for his very significant contribution to organic chemistry in Olomouc.
