# Supplementary material for: Synthesis of Imidazo[2,1‑a]isoindolones via Rearrangement and Tandem Cyclization of Amino-Acid-Based N‑Phenacyl-2-cyano-4-nitrobenzensulfonamides
Source: J Org Chem. 2025 May 27;90(22):7151–60. doi: 10.1021/acs.joc.4c03113 (PMC12150327; doi:10.1021/acs.joc.4c03113)

Supporting Information

Synthesis of Imidazo[2,1-*a*]isoindolones *via* Rearrangement and Tandem Cyclization of Amino Acid-based *N*-Phenacyl-2-cyano-4-nitrobenzensulfonamides

Barbora Lemrová, Kateřina Žáková and Miroslav Soural *

Department of Organic Chemistry, Faculty of Science, Palacký University, 771 46 Olomouc, Czech Republic

*Corresponding author. E-mail: miroslav.soural@upol.cz

Table of Contents:

NMR spectra of final compounds…………………………………………………………………………………S2-S20

(*S*)-3-methyl-5-(4-methylbenzoyl)-8-nitro-1*H*-imidazo[2,1-*a*]isoindol-2(3*H*)-one (5b)

^{1}H NMR (400Mhz; DMSO-D6) spectrum of **5b**

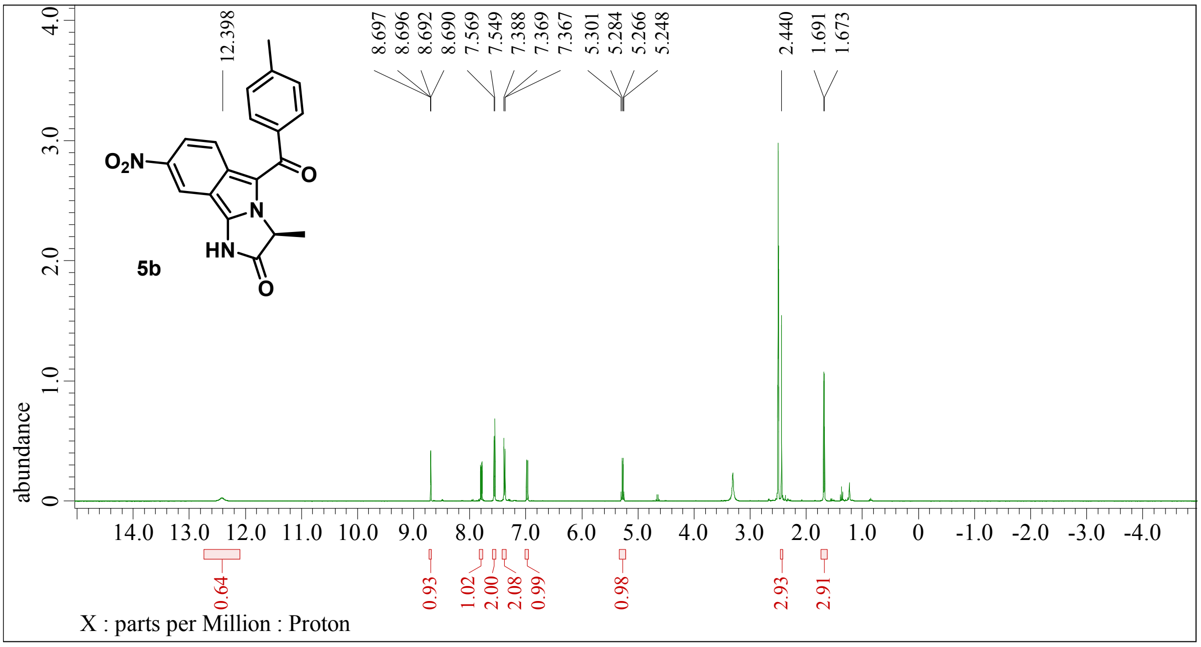

^{13}C{^{1}H} NMR (101 MHz; DMSO-D6) spectrum of **5b**

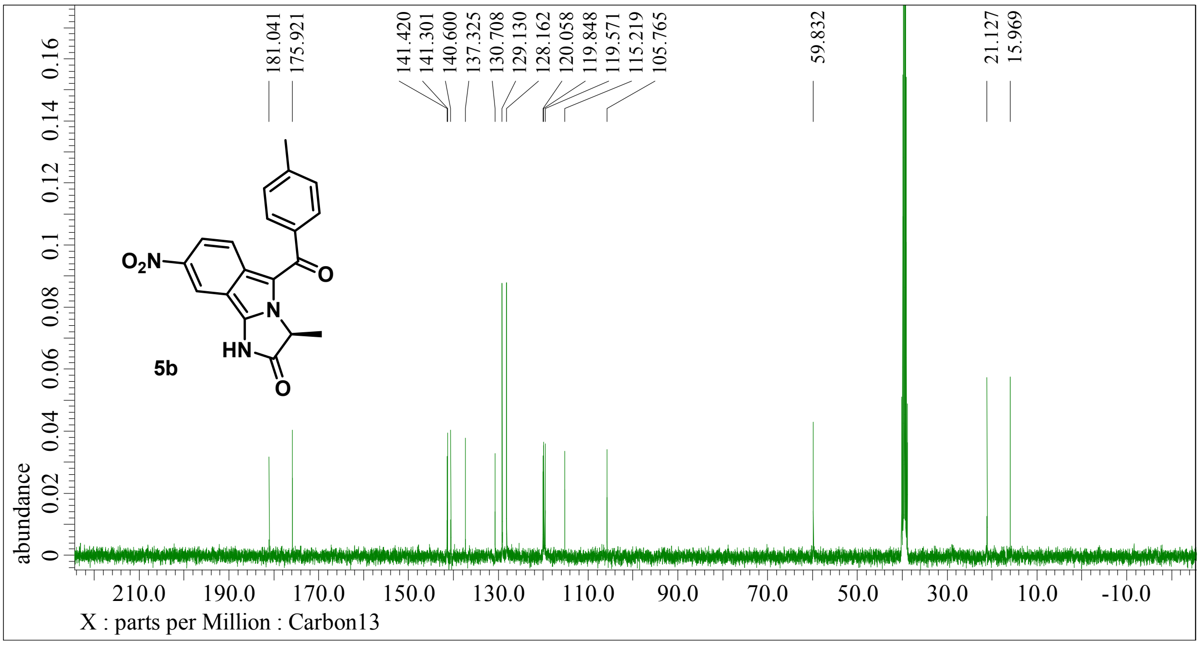

(*S*)-3-benzyl-5-(4-methylbenzoyl)-8-nitro-1*H*-imidazo[2,1-*a*]isoindol-2(3*H*)-one (5c)

^{1}H NMR (400 MHz, CHLOROFORM-D) spectrum of **5c**

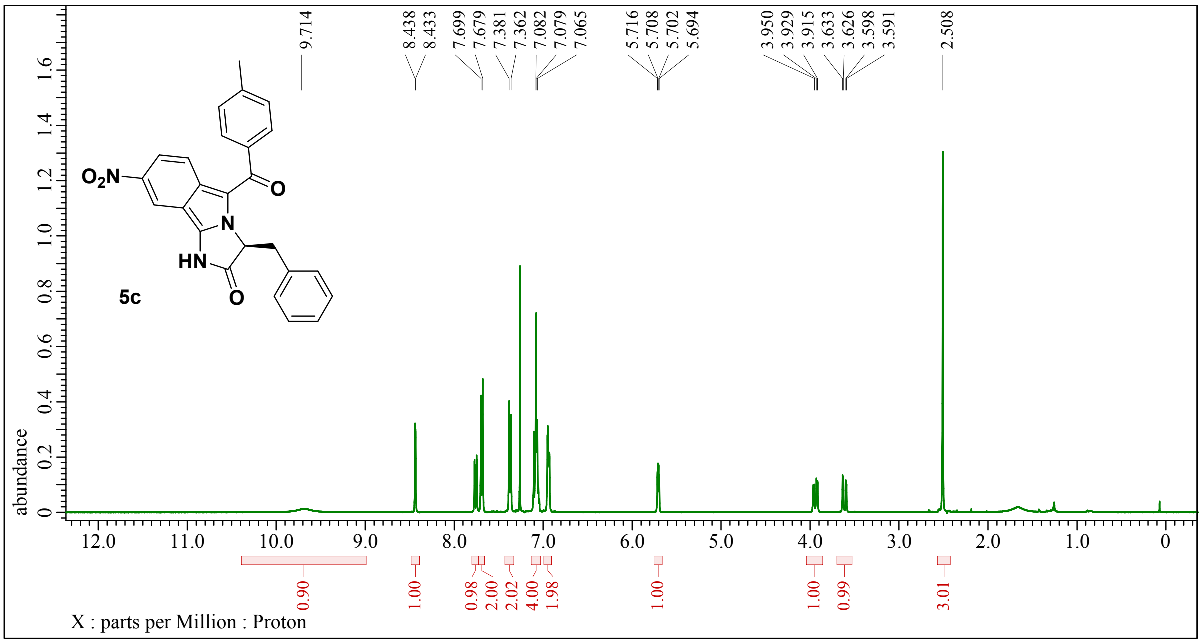

$^{13}C\{^{1}H\}$ NMR (101 MHz; CHLOROFORM-D) spectrum of **5c**

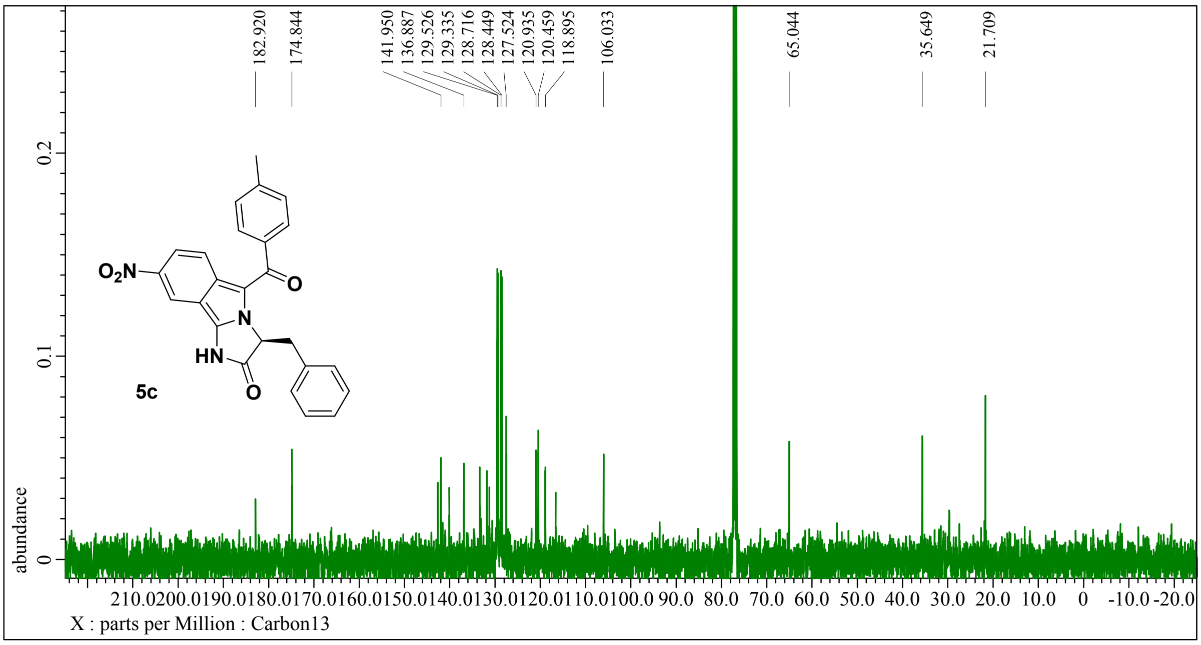

tert-butyl (_S_)-(4-(5-(4-methylbenzoyl)-8-nitro-2-oxo-2,3-dihydro-1_H_-imidazo[2,1-_a_]isoindol-3-yl)butyl)carbamate (5d)

^{1}H NMR (400Mhz; DMSO-D6) spectrum of **5d**

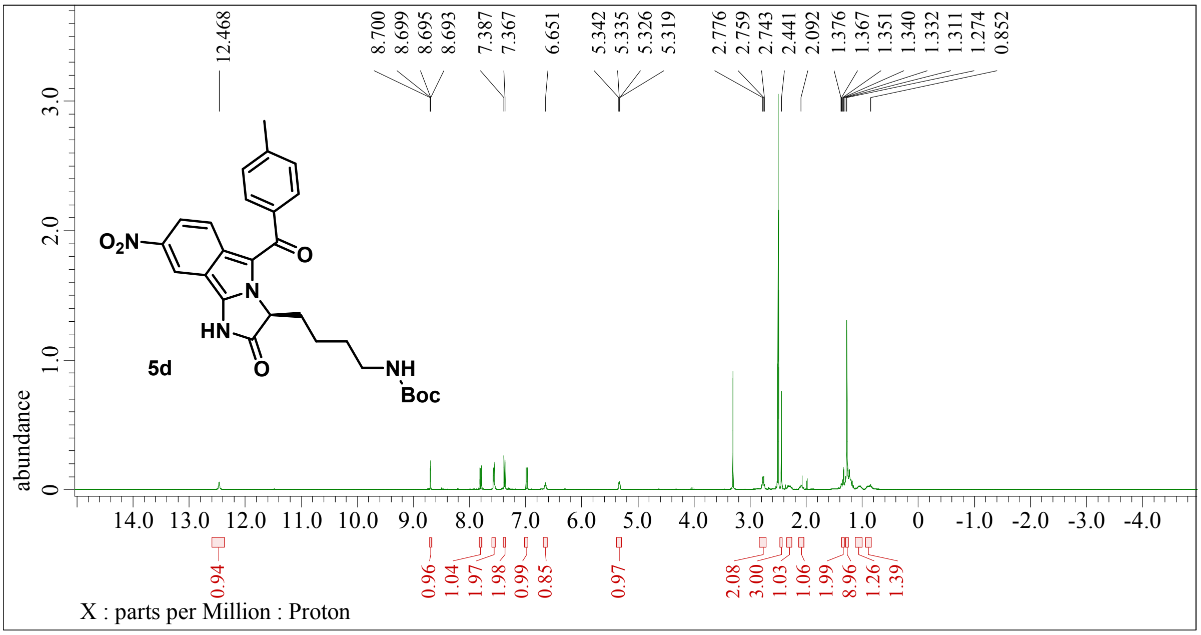

^{13}C{^{1}H} NMR (101 MHz; DMSO-D6) spectrum of **5d**

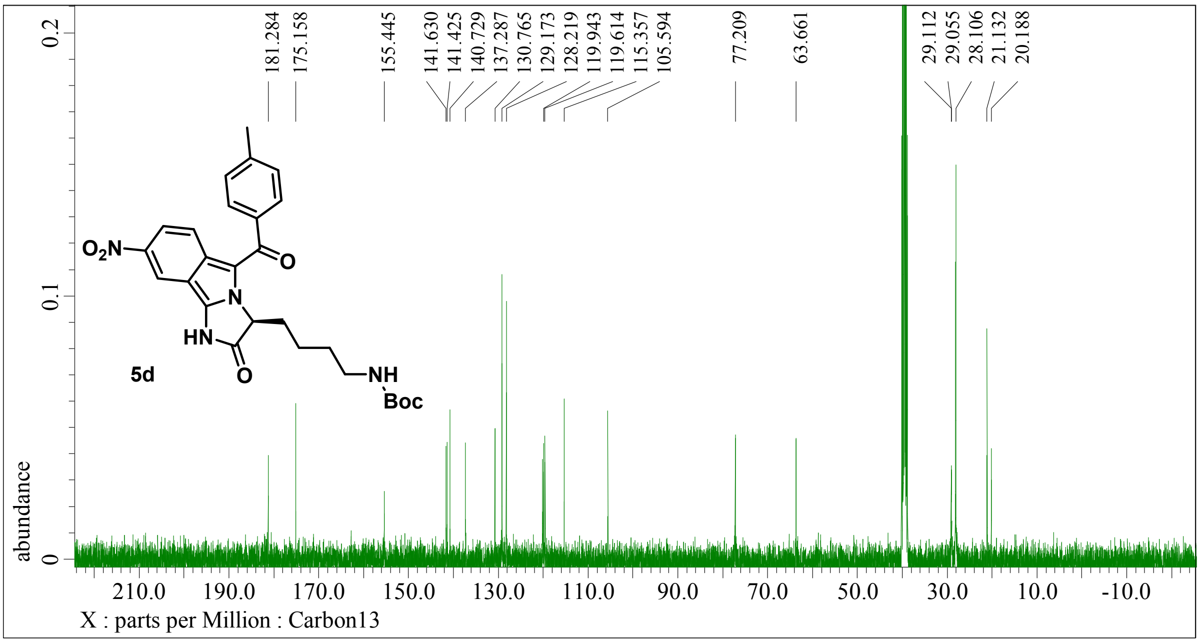

(*S*)-3-(4-(*tert*-butoxy)benzyl)-5-(4-methylbenzoyl)-8-nitro-1*H*-imidazo[2,1-*a*]isoindol-2(3*H*)-one (5e)

^{1}H NMR (400Mhz; CHLOROFORM-D) spectrum of **5e**

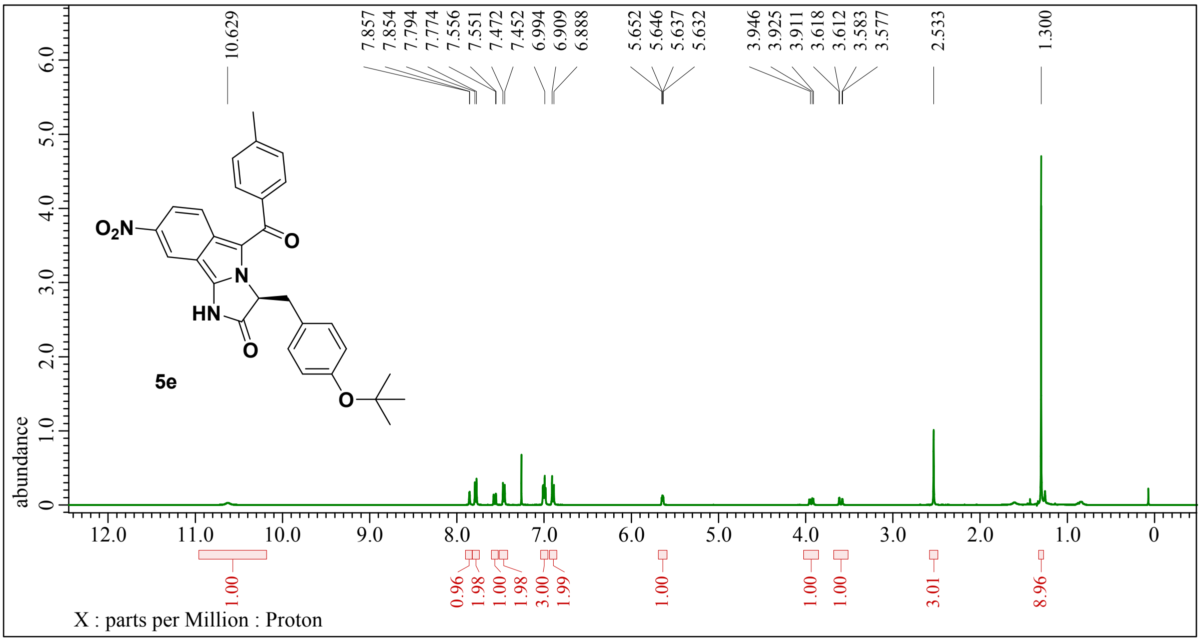

^{13}C{^{1}H} NMR (101 MHz; CHLOROFORM-D) spectrum of **5e**

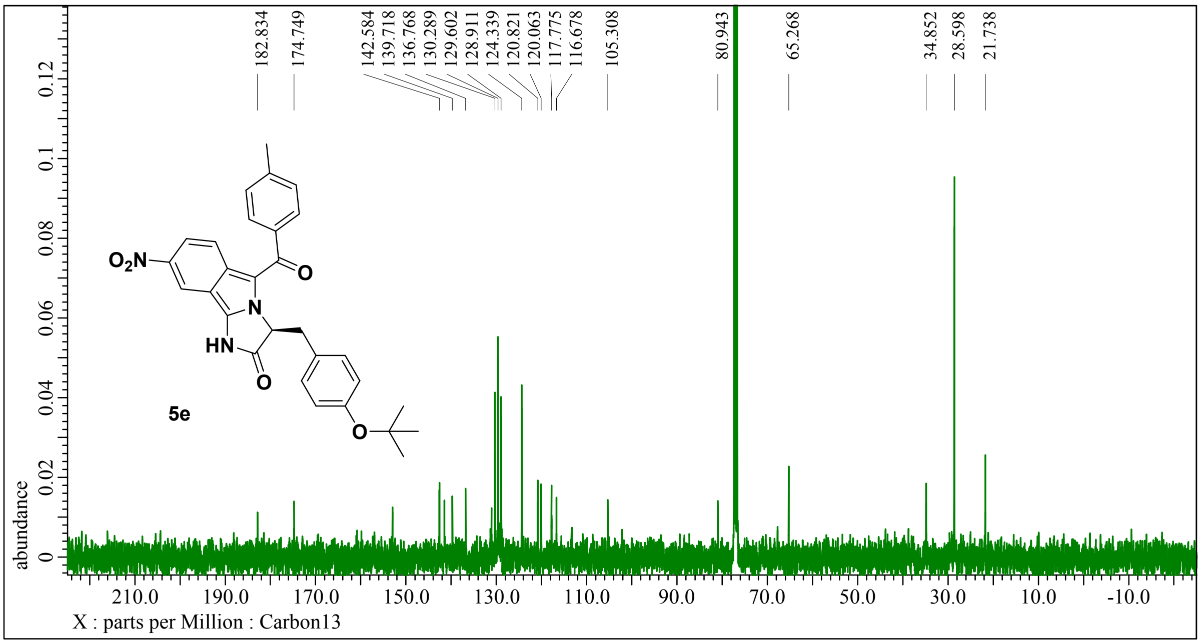

tert-butyl (S)-3-(5-(4-methylbenzoyl)-8-nitro-2-oxo-2,3-dihydro-1H-imidazo[2,1-a]isoindol-3-yl)propanoate (5f)

^{1}H NMR (400Mhz; CHLOROFORM-D) spectrum of **5f**

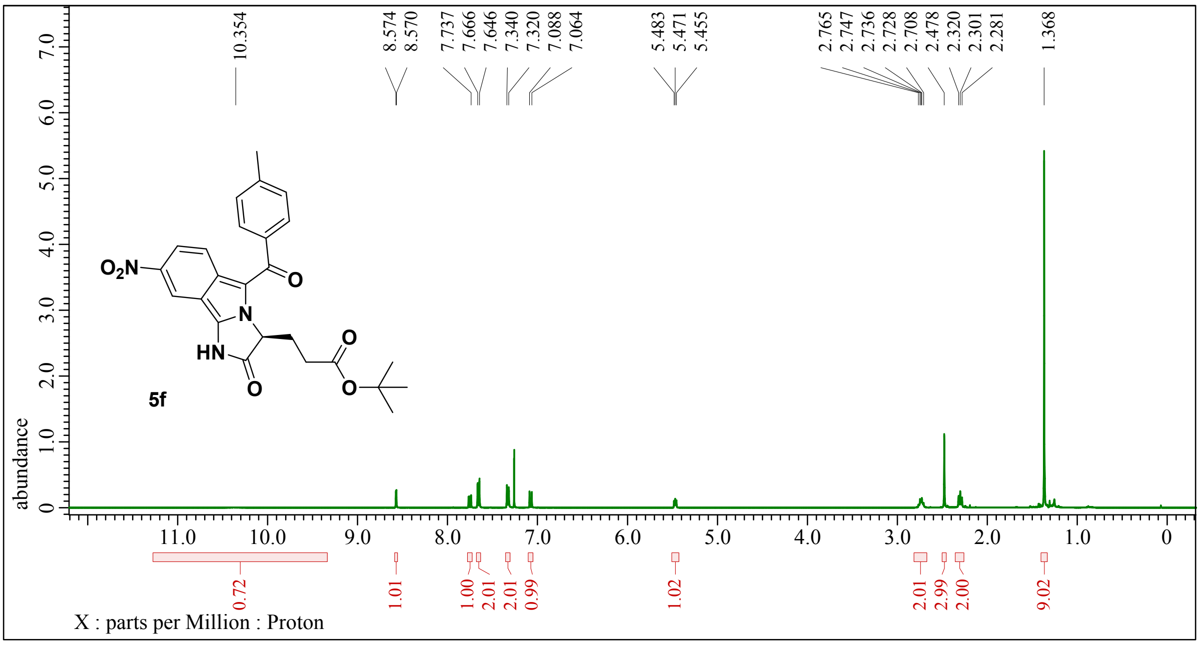

^{13}C{^{1}H} NMR (101 MHz; CHLOROFORM-D) spectrum of **5f**

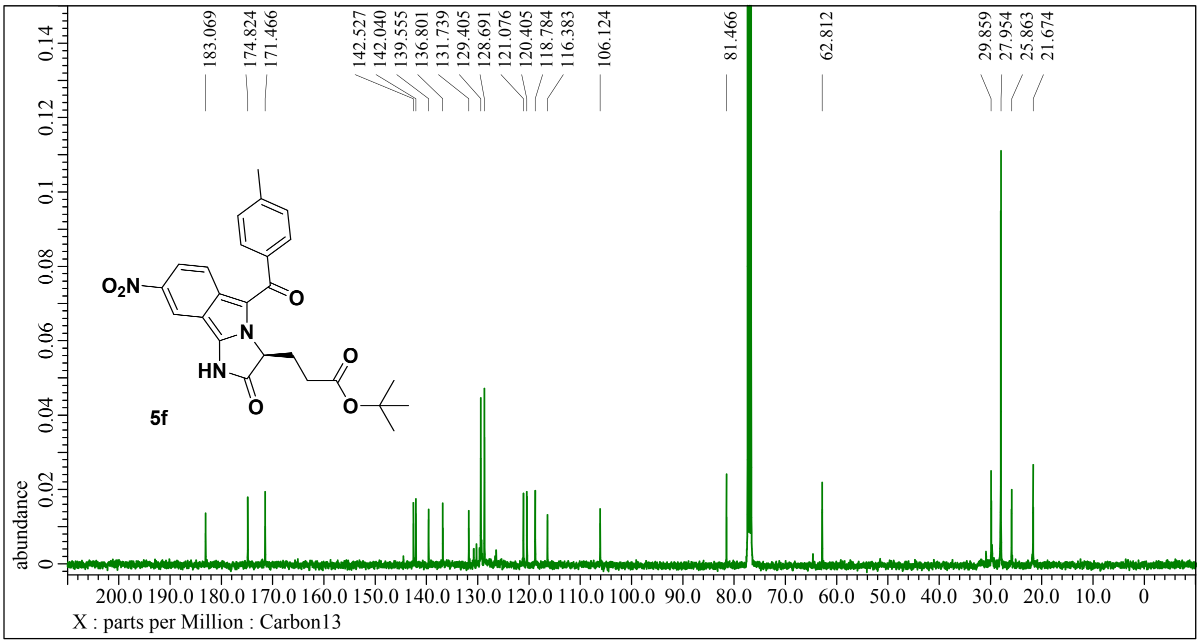

(*S*)-3-isobutyl-5-(4-methylbenzoyl)-8-nitro-1*H*-imidazo[2,1-*a*]isoindol-2(3*H*)-one (5g)

^{1}H NMR (400Mhz; CHLOROFORM-D) spectrum of **5g**

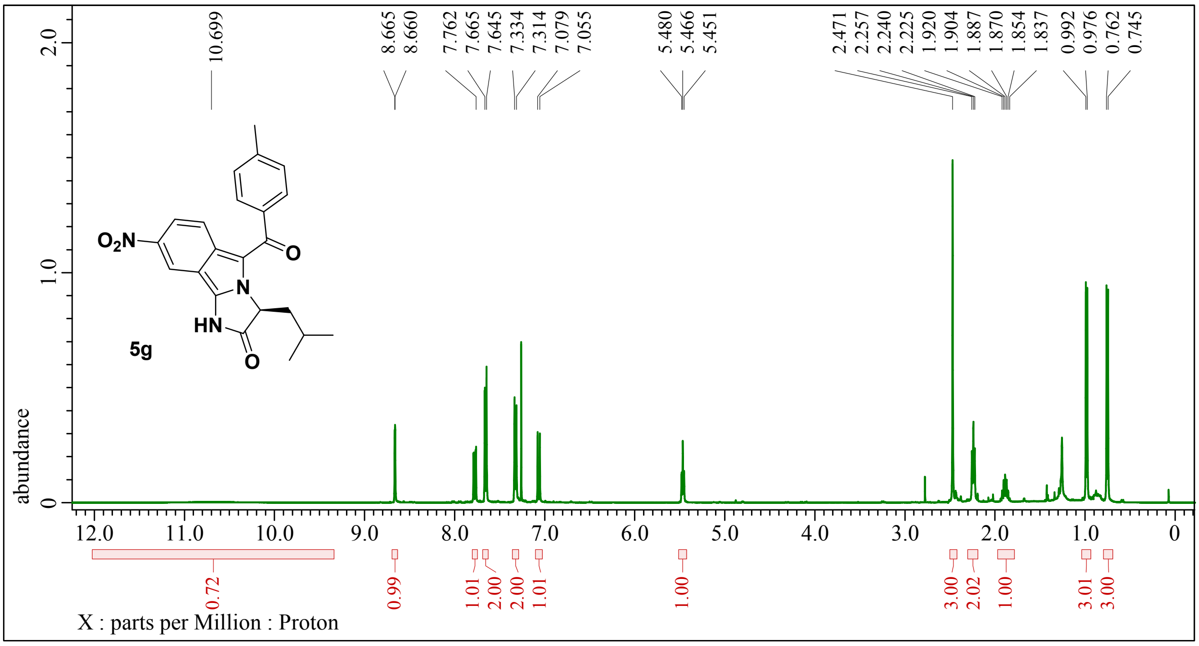

^{13}C{^{1}H} NMR (101 MHz; CHLOROFORM-D) spectrum of **5g**

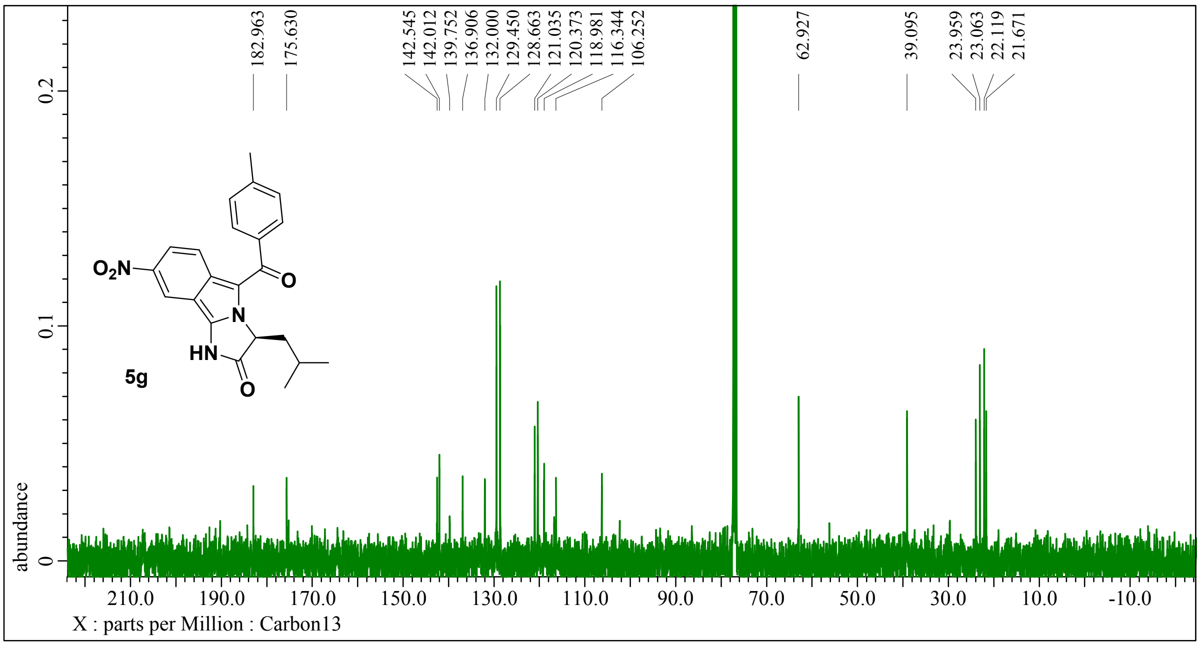

(*S*)-3-(2-azidoethyl)-5-(4-methylbenzoyl)-8-nitro-1*H*-imidazo[2,1-*a*]isoindol-2(3*H*)-one (5h)

^{1}H NMR (400Mhz; DMSO-D6) spectrum of **5h**

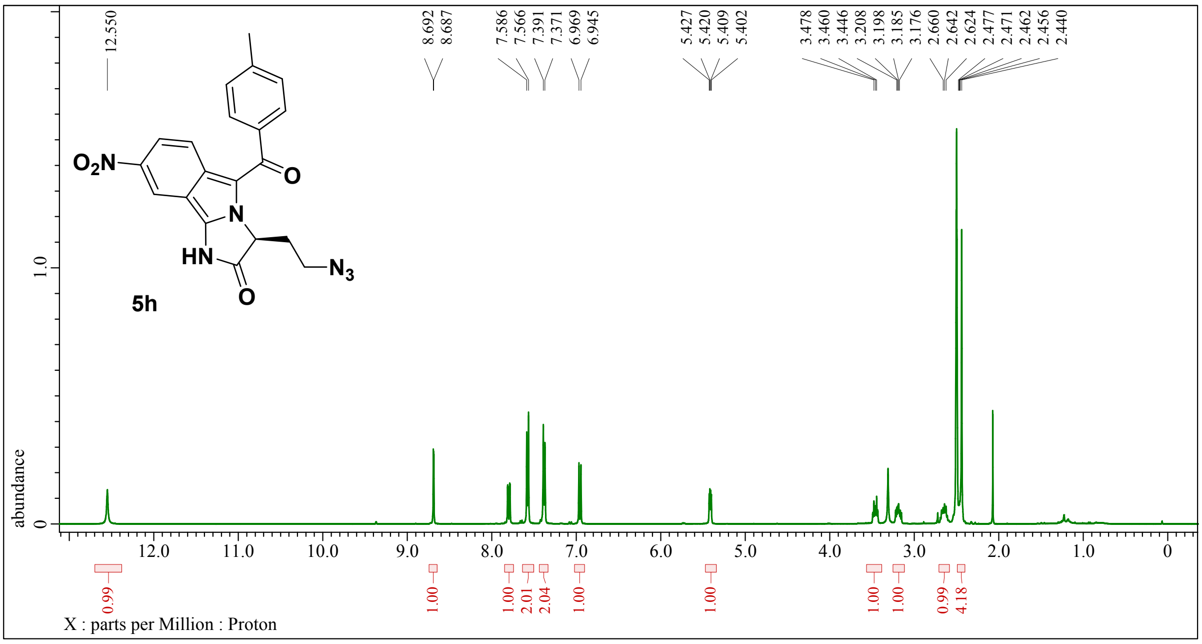

^{13}C{^{1}H} NMR (101 MHz; DMSO-D6) spectrum of **5h**

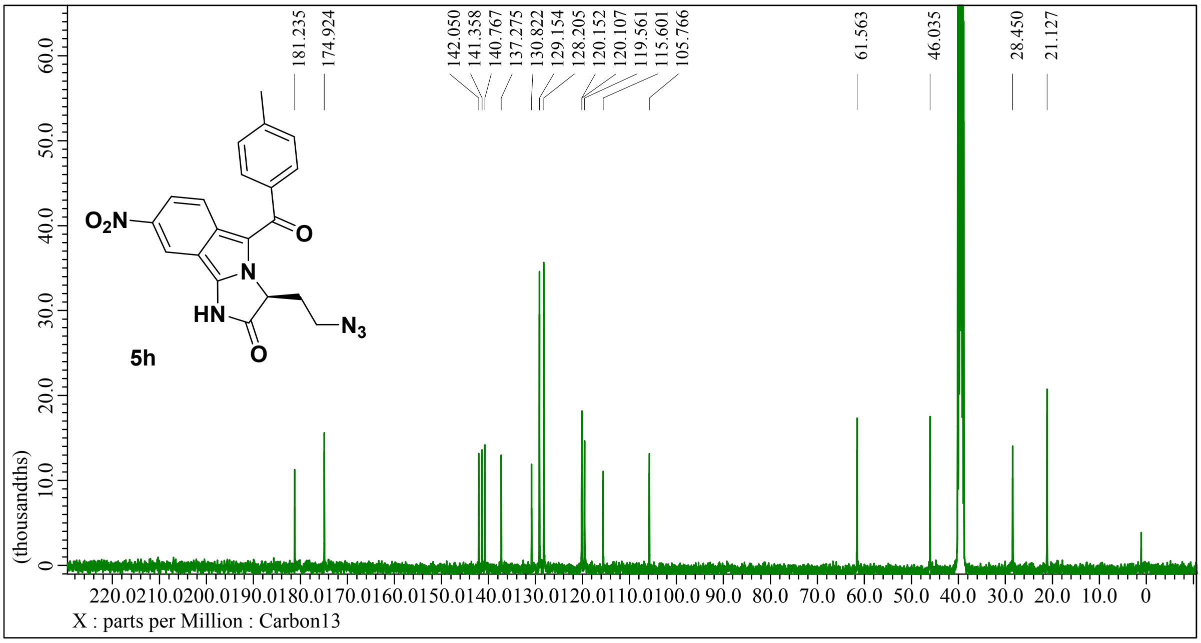

***tert*-butyl (*S*)-3-((5-(4-methylbenzoyl)-8-nitro-2-oxo-2,3-dihydro-1*H*-imidazo[2,1-*a*]isoindol-3-yl)methyl)-1*H*-indole-1-carboxylate (5i)**

^{1}H NMR (400Mhz; CHLOROFORM-D) spectrum of **5i**

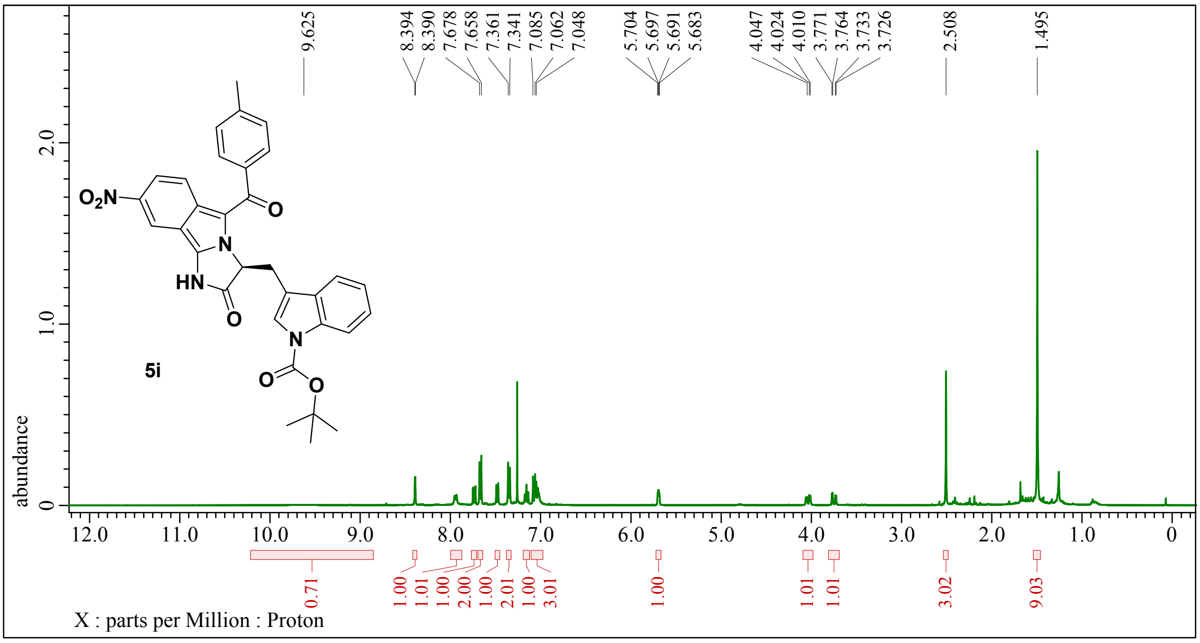

$^{13}C\{^{1}H\}$ NMR (101 MHz; CHLOROFORM-D) spectrum of **5i**

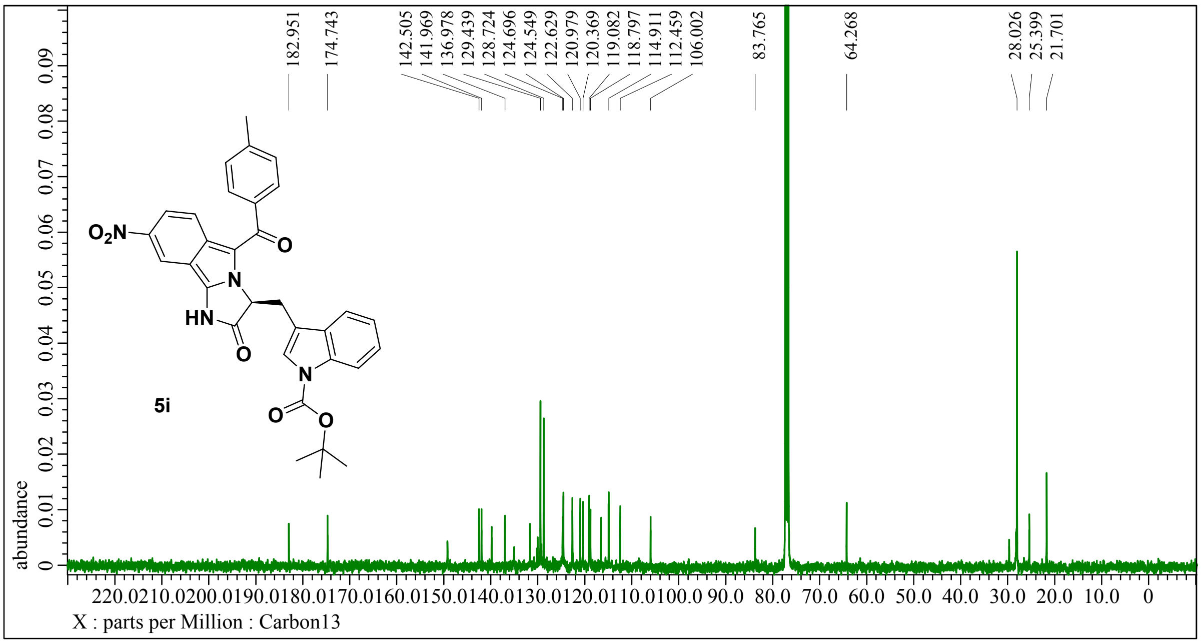

(*S*)-2,2,4,6,7-pentamethyl-*N*-(*N*-(3-(5-(4-methylbenzoyl)-8-nitro-2-oxo-2,3-dihydro-1*H*-imidazo[2,1-*a*]isoindol-3-yl)propyl)carbamimidoyl)-2,3-dihydrobenzofuran-5-sulfonamide (5j)

^{1}H NMR (400Mhz; CHLOROFORM-D) spectrum of **5j**

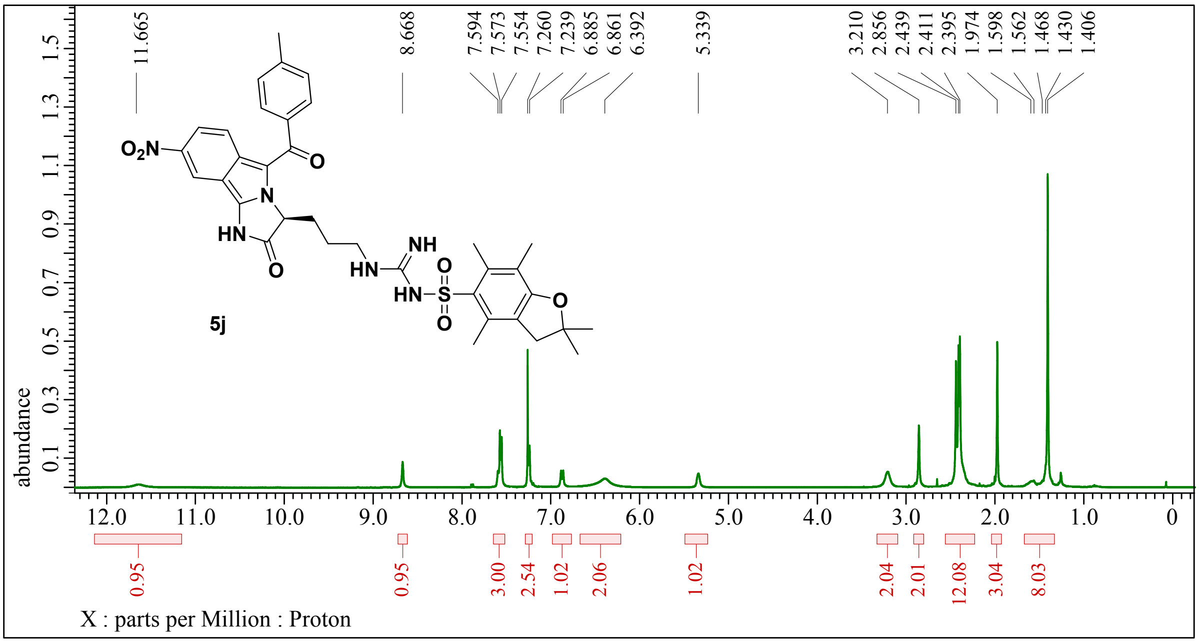

^{13}C{^{1}H} NMR (101 MHz; CHLOROFORM-D) spectrum of **5j**

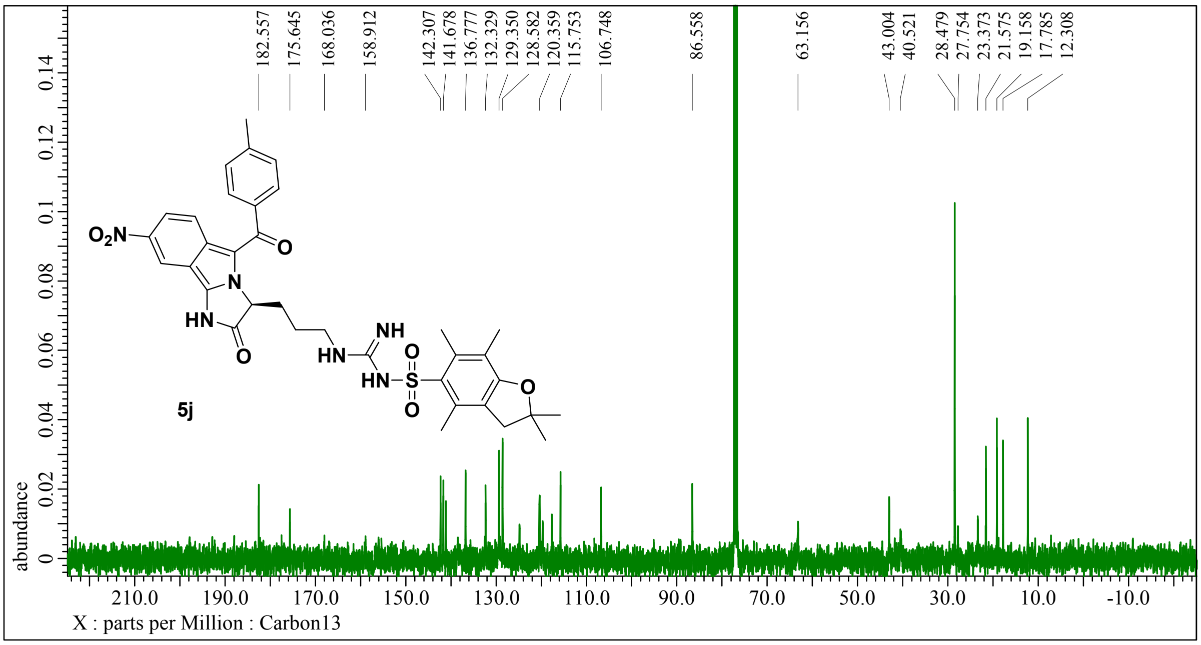

(*S*)-5-(4-chlorobenzoyl)-3-methyl-8-nitro-1*H*-imidazo[2,1-*a*]isoindol-2(3*H*)-one (5k)

^{1}H NMR (400Mhz; DMSO-D6) spectrum of **5k**

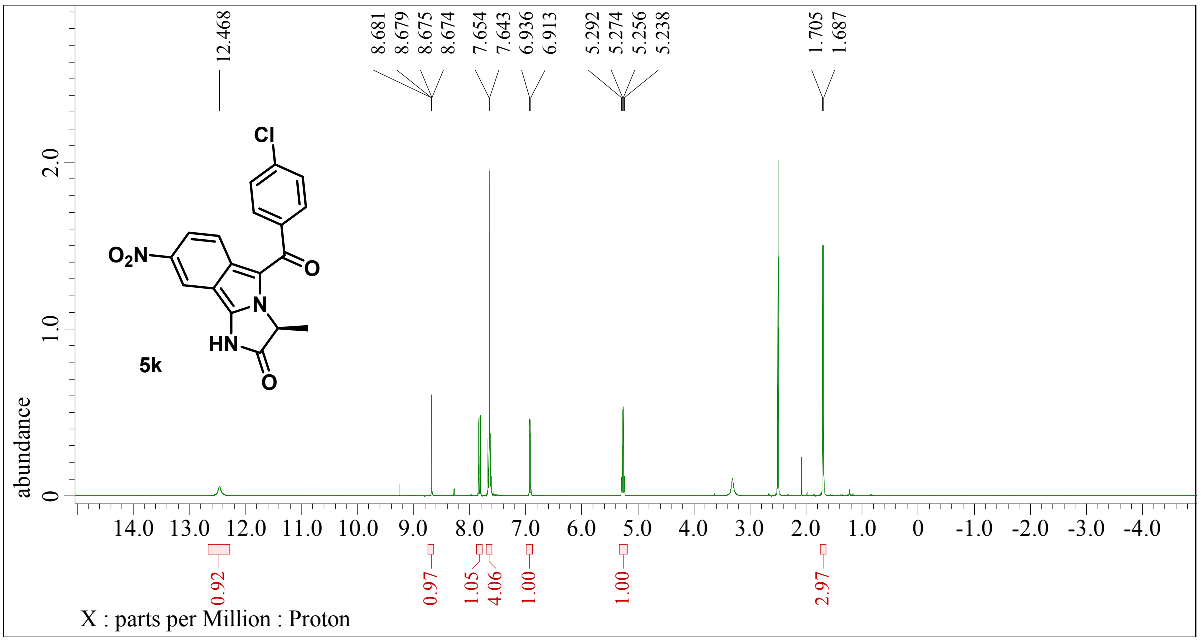

$^{13}C\{^{1}H\}$ NMR (101 MHz; DMSO-D6) spectrum of **5k**

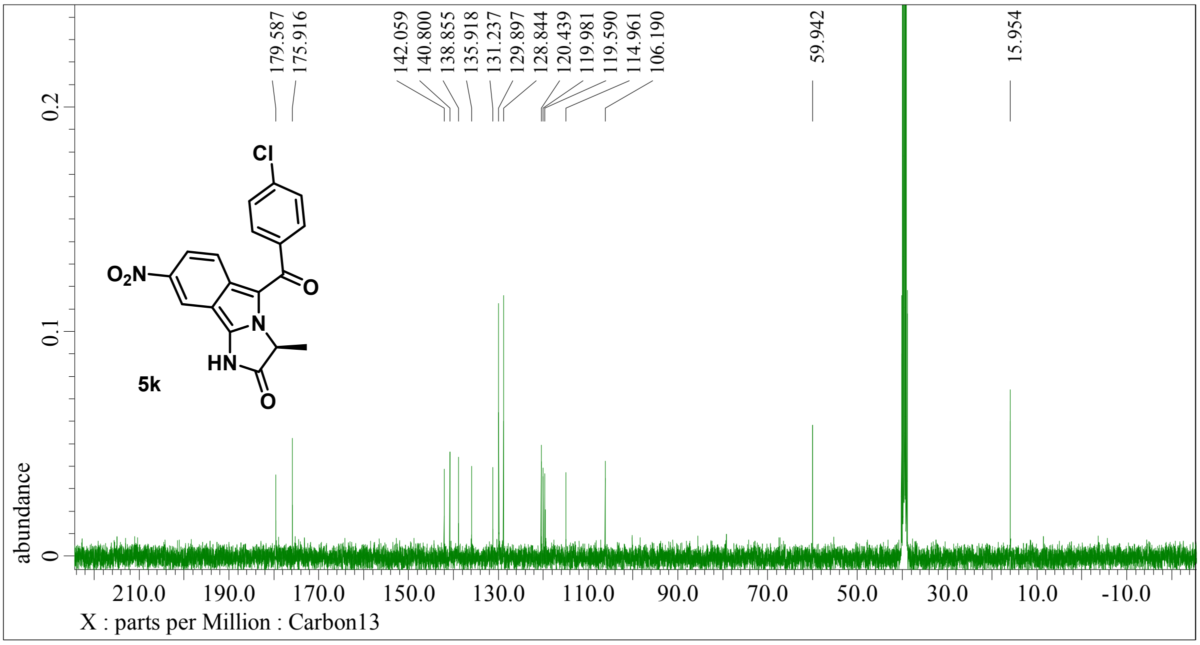

(*S*)-3-methyl-5-(2-methylbenzoyl)-8-nitro-1*H*-imidazo[2,1-*a*]isoindol-2(3*H*)-one (5l)

^{1}H NMR (400Mhz; DMSO-D6) spectrum of **5l**

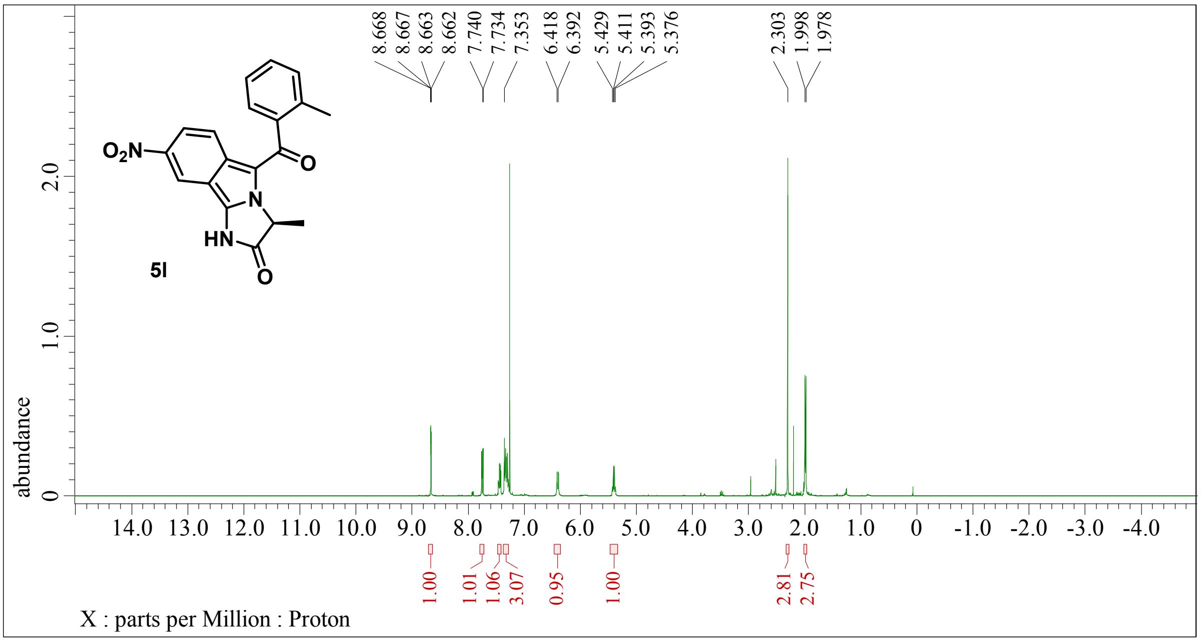

^{13}C{^{1}H} NMR (101 MHz; DMSO-D6) spectrum of **5l**

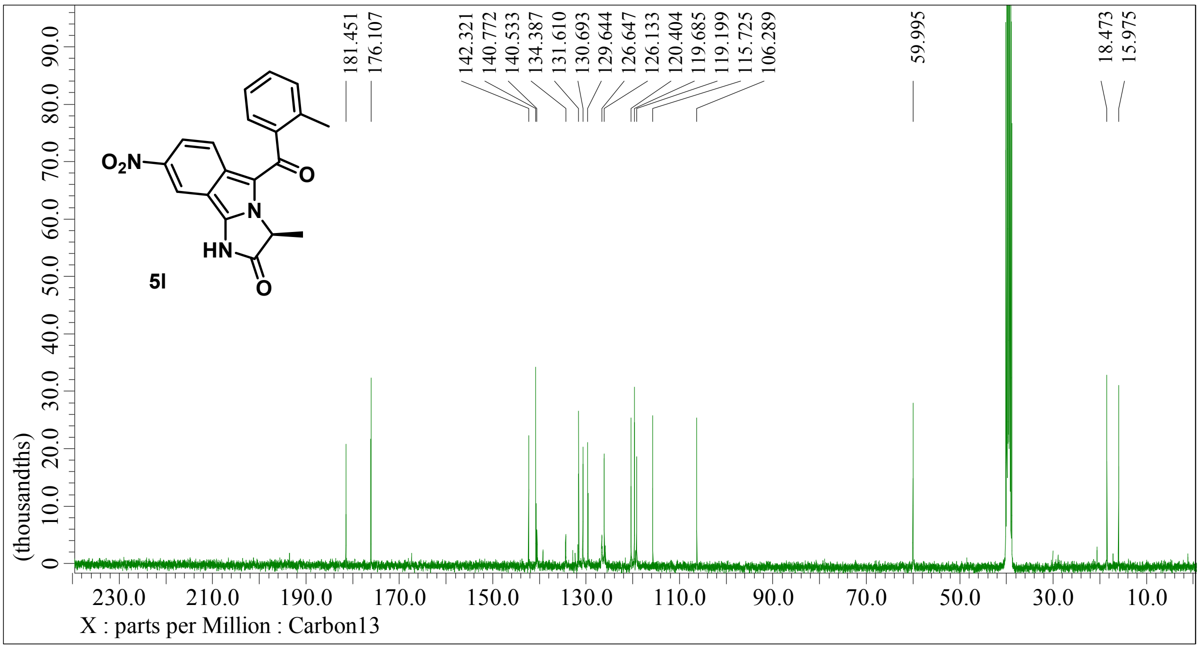

(*S*)-5-(4-amino-3,5-dichlorobenzoyl)-3-methyl-8-nitro-1*H*-imidazo[2,1-*a*]isoindol-2(3*H*)-one (5m)

^{1}H NMR (400Mhz; DMSO-D6) spectrum of **5m**

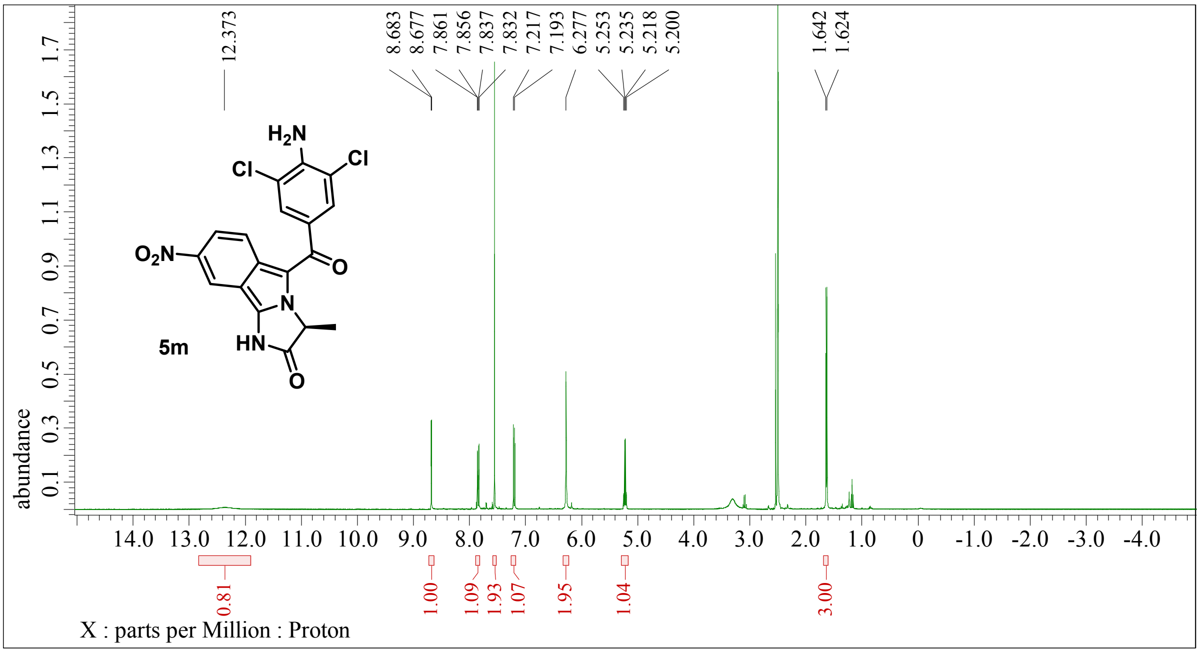

^{13}C{^{1}H} NMR (101 MHz; DMSO-D6) spectrum of **5m**

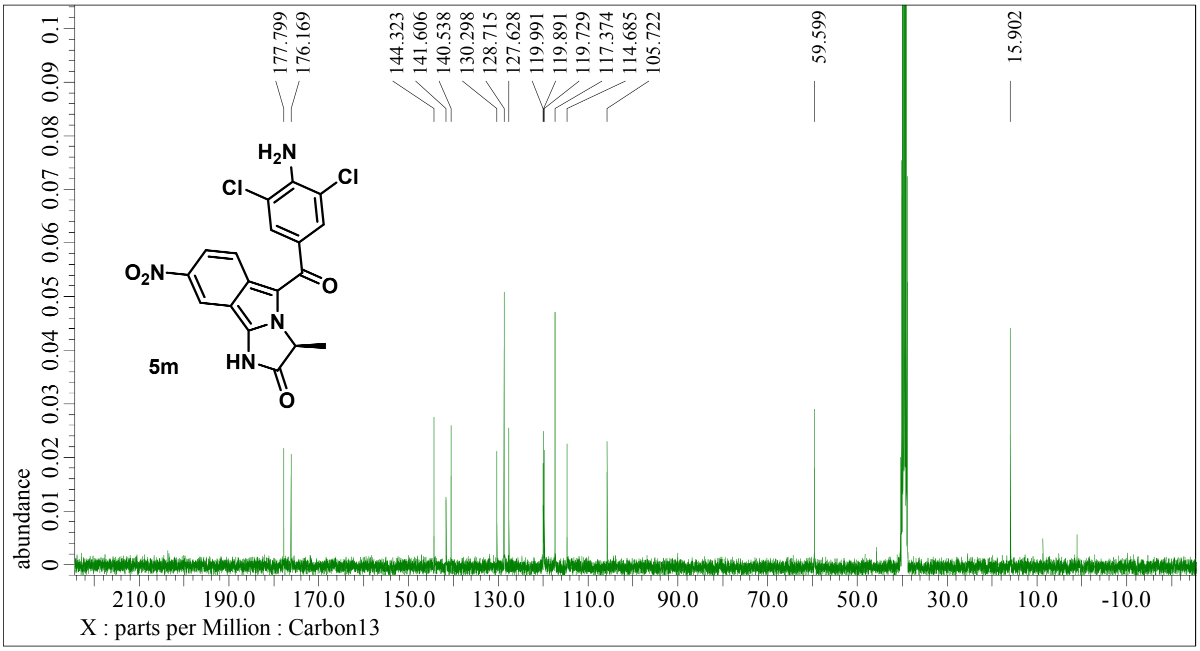

(*S*)-5-(4-methoxybenzoyl)-3-methyl-8-nitro-1*H*-imidazo[2,1-*a*]isoindol-2(3*H*)-one (5n)

^{1}H NMR (400Mhz; DMSO-D6) spectrum of **5n**

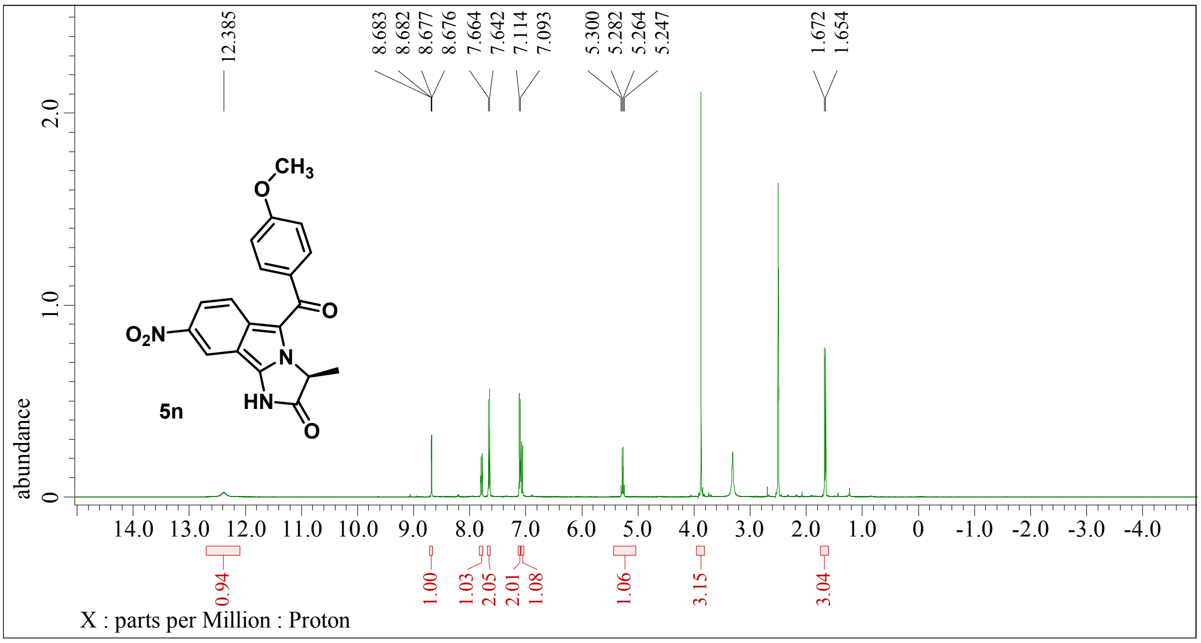

^{13}C{^{1}H} NMR (101 MHz; DMSO-D6) spectrum of **5n**

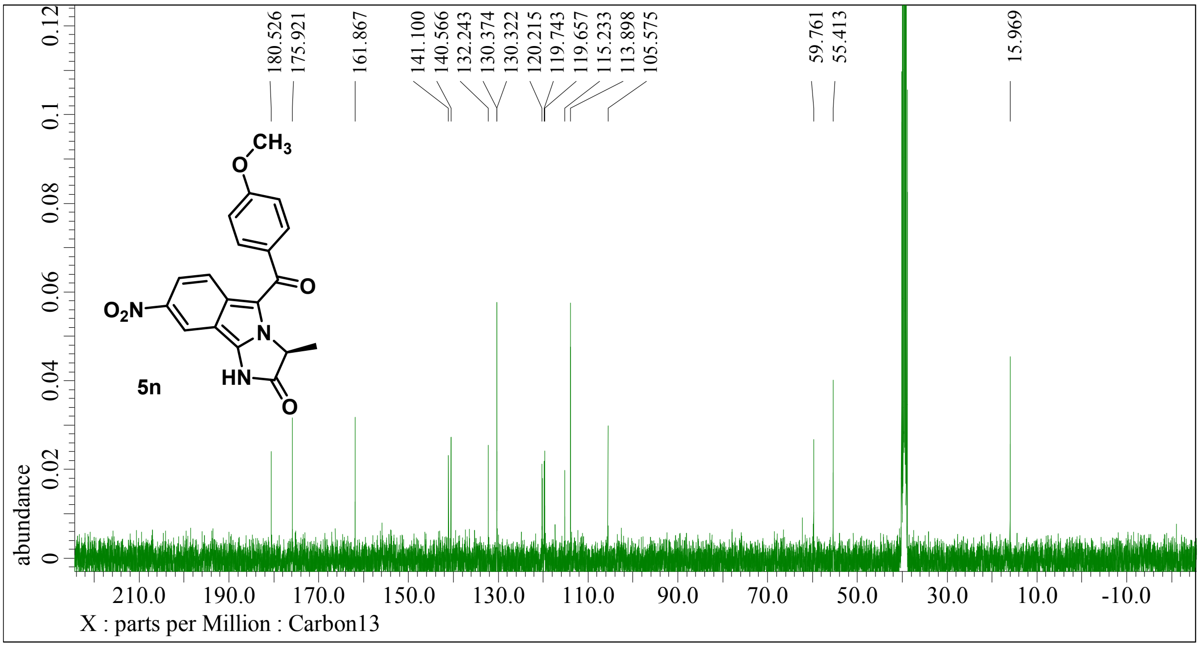

(*S*)-5-(4-fluorobenzoyl)-3-methyl-8-nitro-1*H*-imidazo[2,1-*a*]isoindol-2(3*H*)-one (5o)

^{1}H NMR (400Mhz; DMSO-D6) spectrum of **5o**

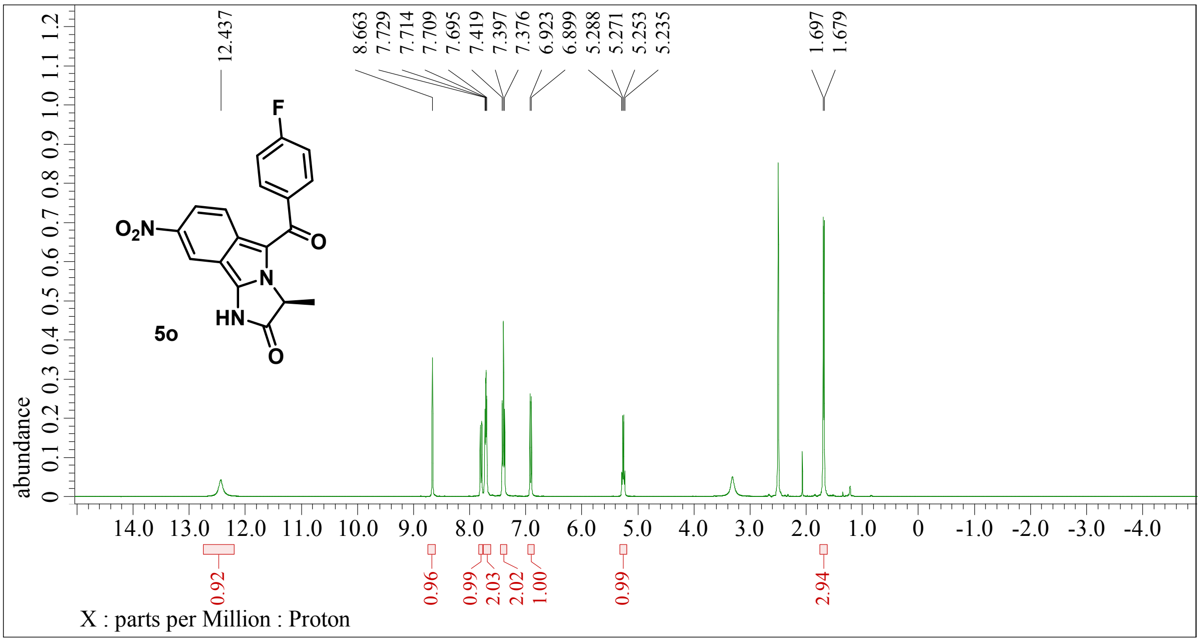

$^{13}C\{^{1}H\}$ NMR (101 MHz; DMSO-D6) spectrum of **5o**

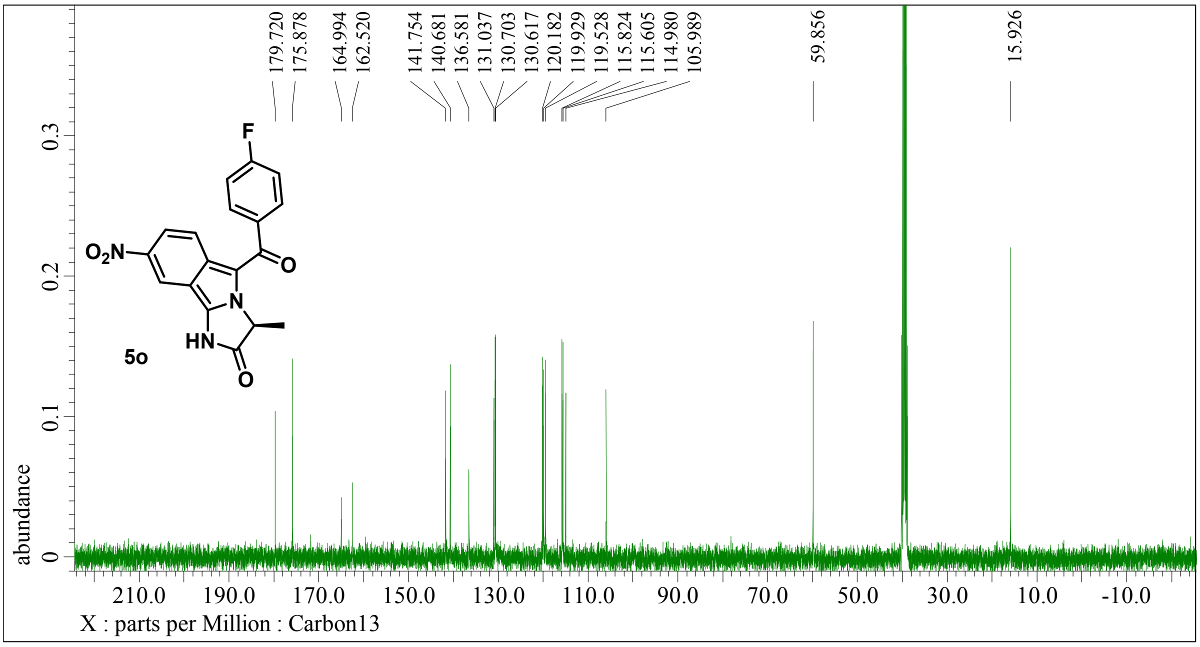

6-(4-methylbenzoyl)-9-nitro-3,4-dihydropyrimido[2,1-*a*]isoindol-2(1*H*)-one (6)

^{1}H NMR (400Mhz; DMSO-D6) spectrum of **6**

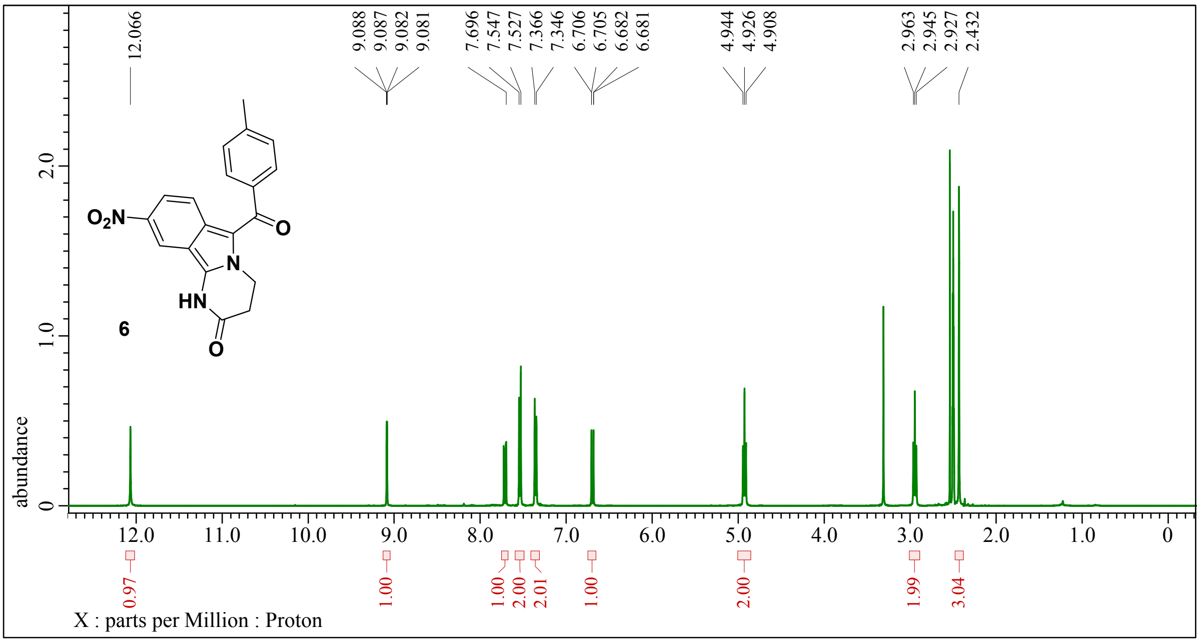

$^{13}C\{^{1}H\}$ NMR (101 MHz; DMSO-D6) spectrum of **6**

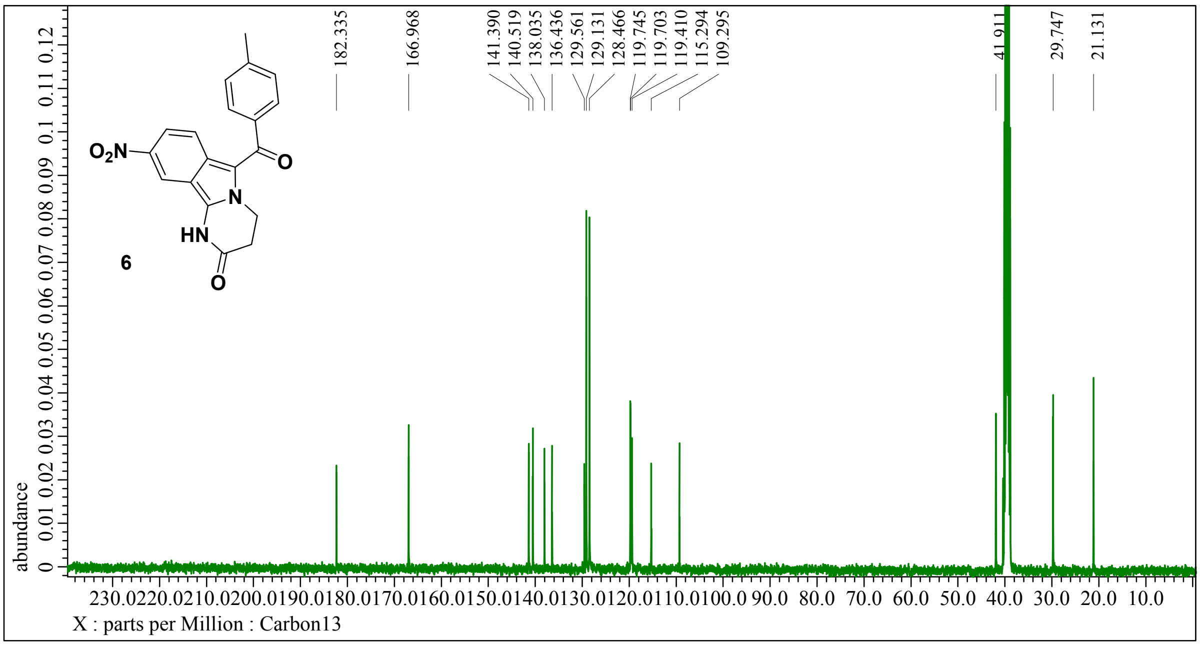

Methyl ((2-cyano-4-nitrophenyl)sulfonyl)-L-alaninate (8b)

^{1}H NMR (400Mhz; DMSO-D6) spectrum of rotamers **8b**

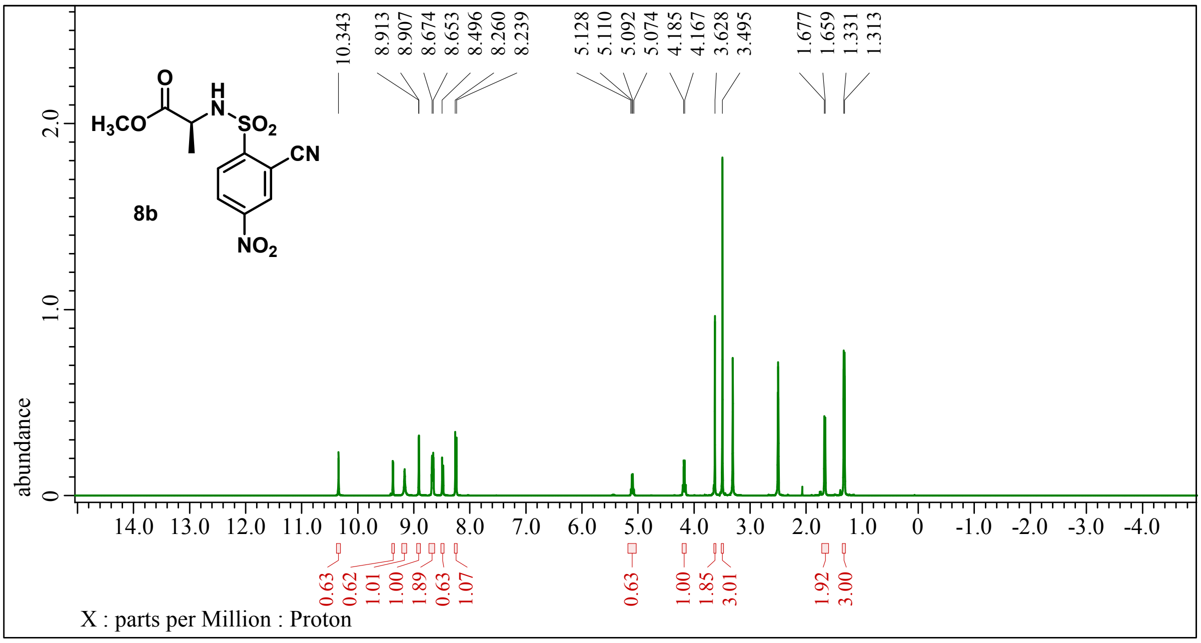

^{13}C{^{1}H} NMR (101 MHz; DMSO-D6) spectrum of rotamers **8b**

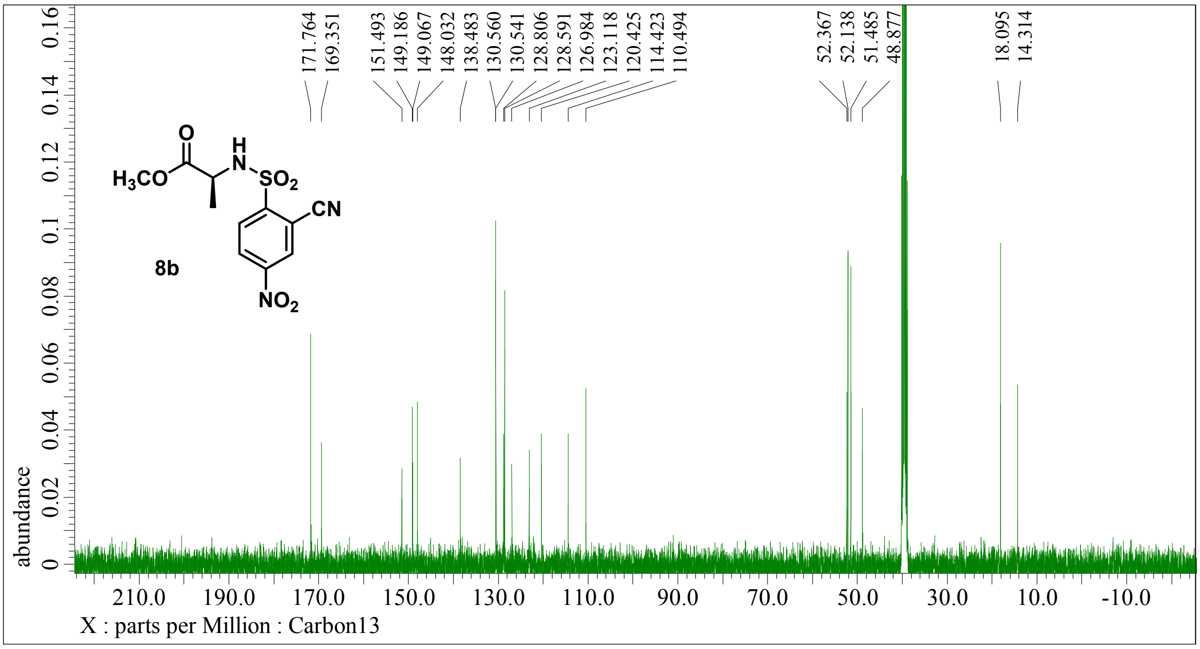

Methyl ((2-cyano-4-nitrophenyl)sulfonyl)-*L*-phenylalaninate (8c)

^{1}H NMR (400Mhz; CHLOROFORM-D) spectrum of **8c**

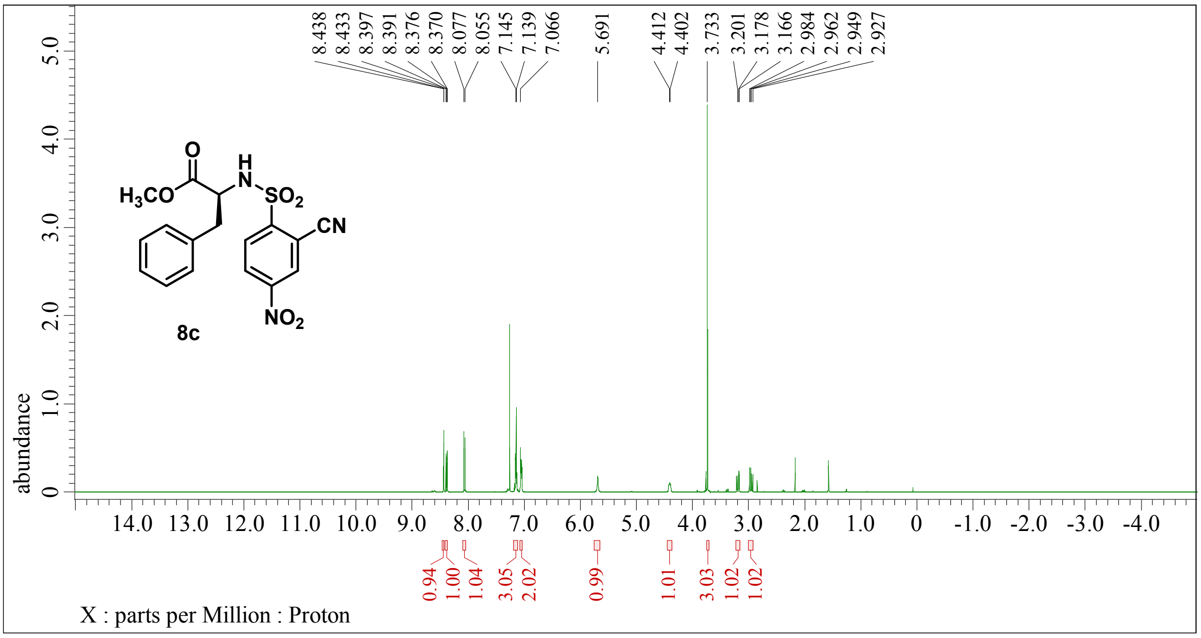

$^{13}C\{^{1}H\}$ NMR (101 MHz; CHLOROFORM-D) spectrum of **8c**

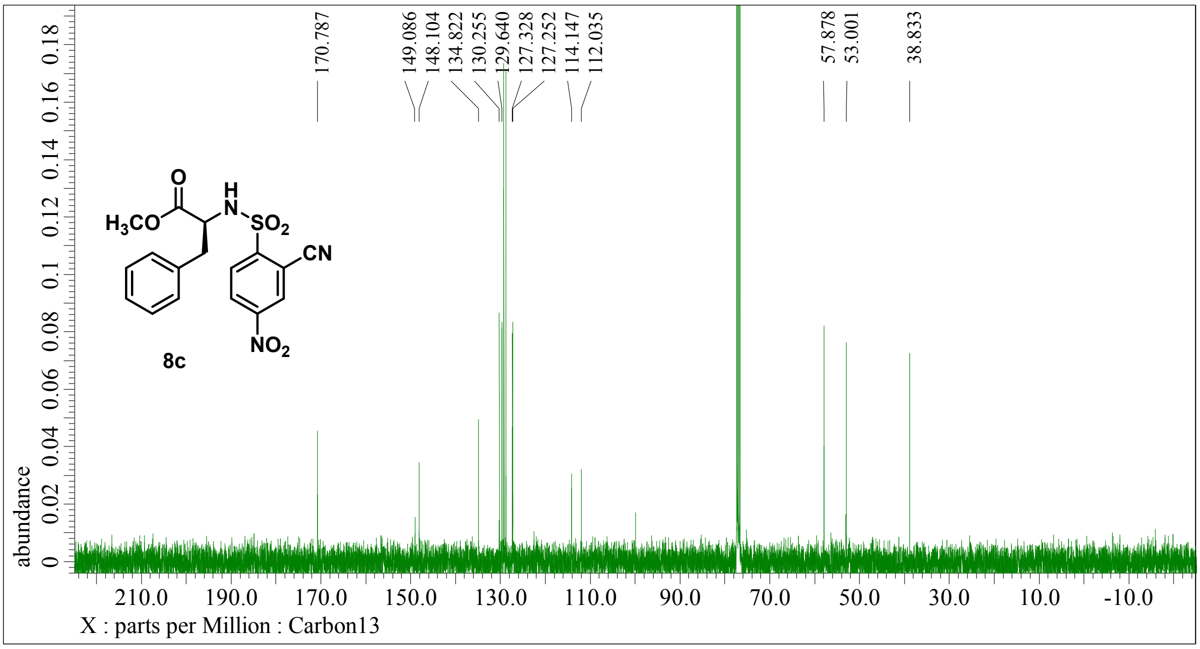

Methyl *N*-((2-cyano-4-nitrophenyl)sulfonyl)-*N*-(2-oxo-2-(p-tolyl)ethyl)-*L*-alaninate (9b)

^{1}H NMR (400Mhz; CHLOROFORM-D) spectrum of **9b**

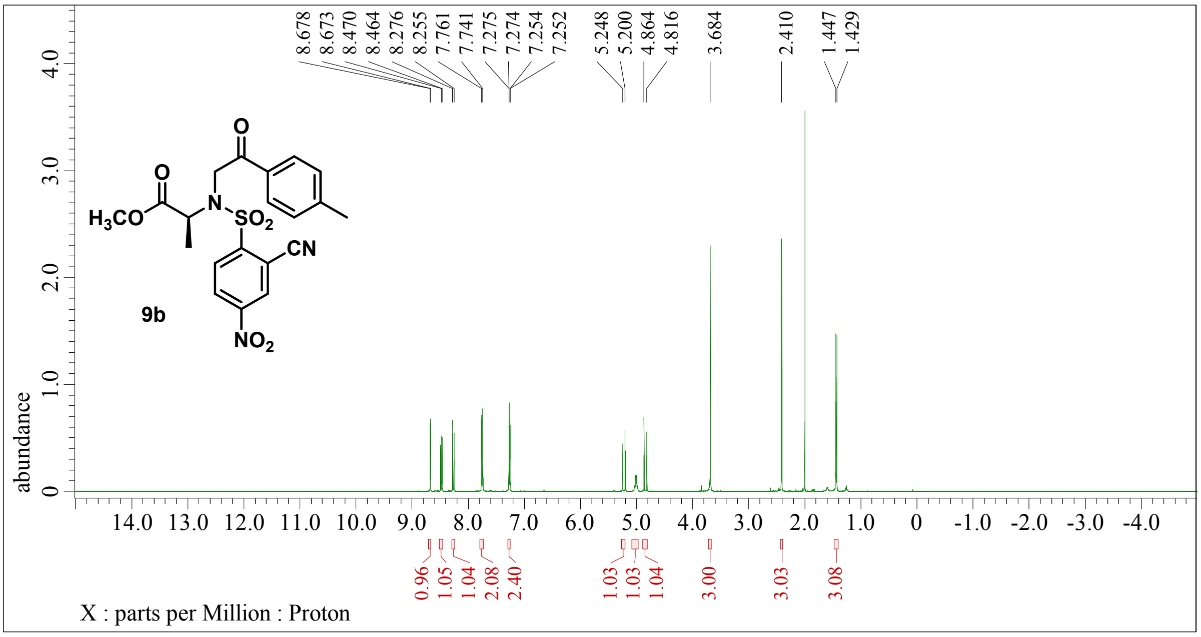

^{13}C{^{1}H} NMR (101 MHz; CHLOROFORM-D) spectrum of **9b**

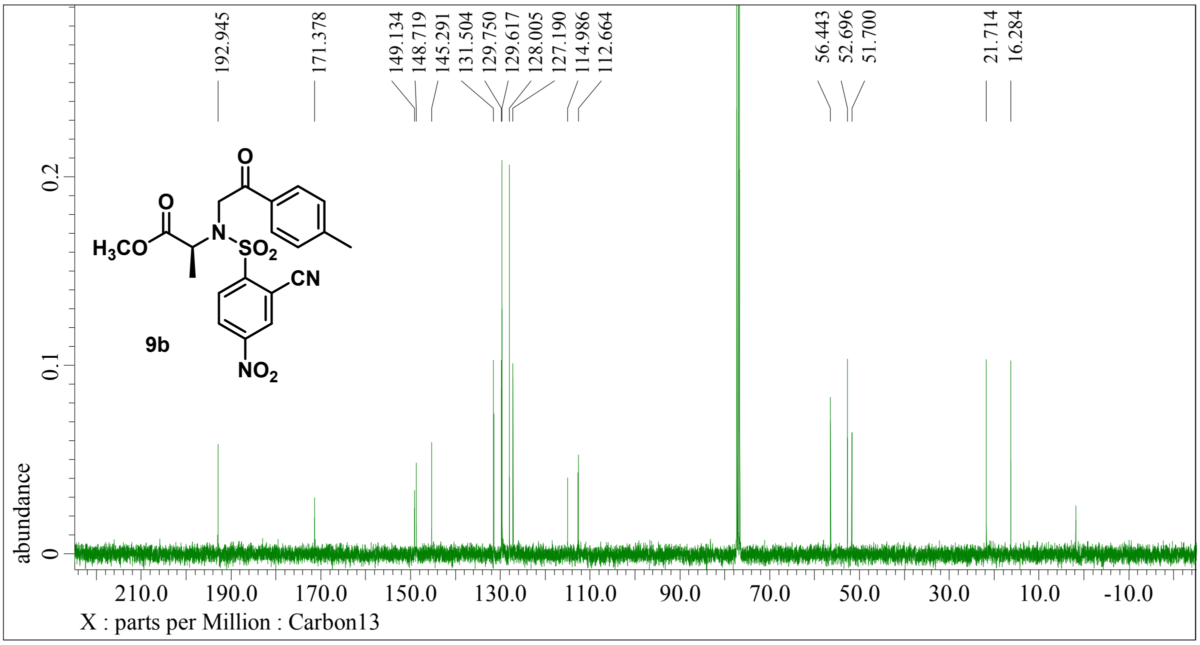

Methyl *N*-((2-cyano-4-nitrophenyl)sulfonyl)-*N*-(2-oxo-2-(*p*-tolyl)ethyl)-*L*-phenylalaninate (9c)

^{1}H NMR (400Mhz; CHLOROFORM-D) spectrum of **9c**

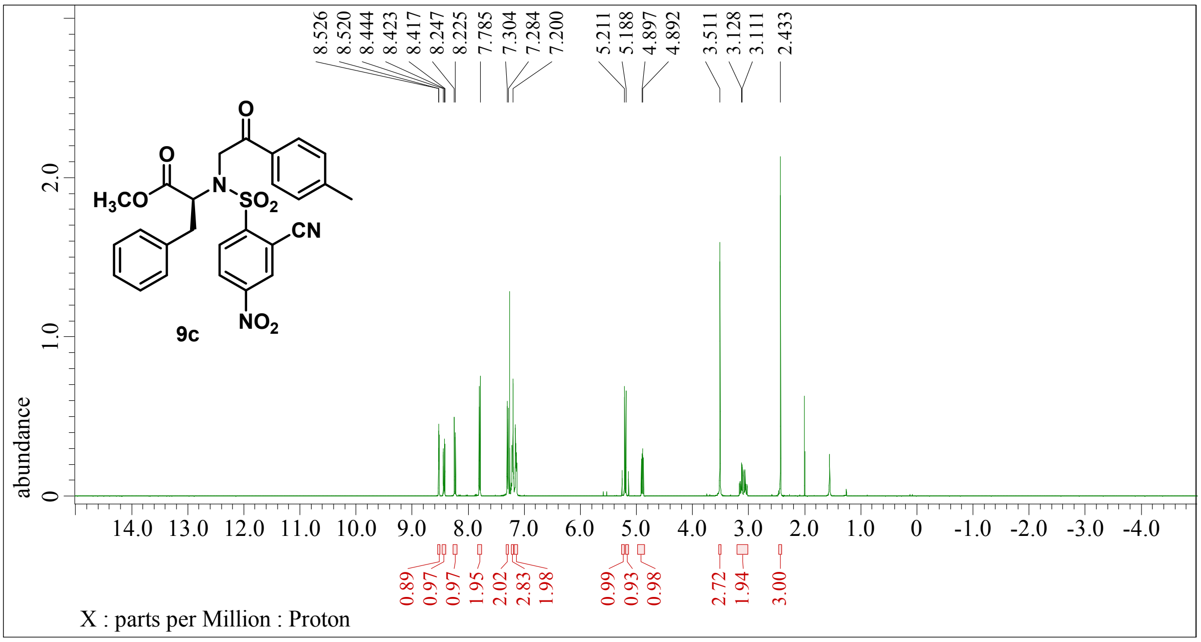

^{13}C{^{1}H} NMR (101 MHz; CHLOROFORM-D) spectrum of **9c**

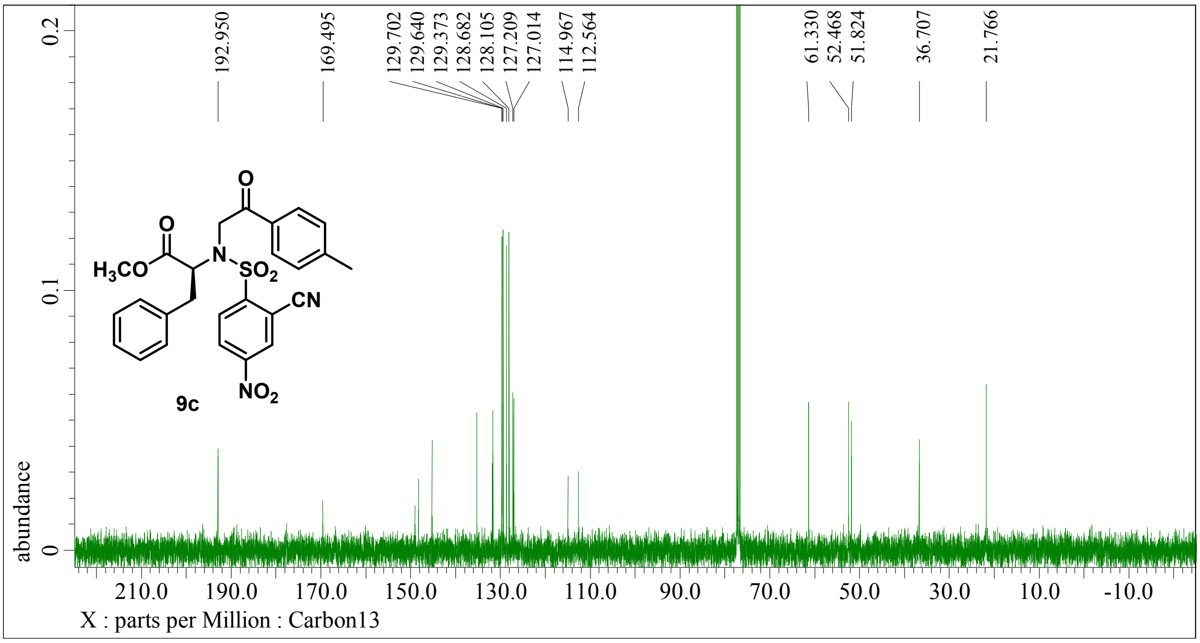

Supplement: Supplementary file 1 [file jo4c03113_si_001.pdf]
